# Advanced brain aging in Parkinson’s disease with cognitive impairment

**DOI:** 10.1038/s41531-024-00673-7

**Published:** 2024-03-16

**Authors:** Chang-Le Chen, Shao-Ying Cheng, Leila Montaser-Kouhsari, Wen-Chao Wu, Yung-Chin Hsu, Chun-Hwei Tai, Wen-Yih Isaac Tseng, Ming-Che Kuo, Ruey-Meei Wu

**Affiliations:** 1 Institute of Medical Device and Imaging, National Taiwan University College of Medicine, Taipei, Taiwan; 2 Department of Bioengineering, University of Pittsburgh, Pittsburgh, PA USA; 3 Department of Neurology, National Taiwan University Hospital Bei-Hu Branch, Taipei, Taiwan; 4 Department of Neurology, Harvard University, Boston, MA USA; 5 Acroviz Inc, Taipei, Taiwan; 6 Department of Neurology, National Taiwan University Hospital, Taipei, Taiwan; 7 Department of Medical Imaging, National Taiwan University Hospital, Taipei, Taiwan; 8 Department of Medicine, National Taiwan University Cancer Center, Taipei, Taiwan; 9 Neurobiology and Cognitive Science Center, National Taiwan University, Taipei, Taiwan

**Keywords:** Parkinson's disease, Diagnostic markers, Diagnostic markers, Parkinson's disease

## Abstract

Patients with Parkinson’s disease and cognitive impairment (PD-CI) deteriorate faster than those without cognitive impairment (PD-NCI), suggesting an underlying difference in the neurodegeneration process. We aimed to verify brain age differences in PD-CI and PD-NCI and their clinical significance. A total of 94 participants (PD-CI, *n* = 27; PD-NCI, *n* = 34; controls, *n* = 33) were recruited. Predicted age difference (PAD) based on gray matter (GM) and white matter (WM) features were estimated to represent the degree of brain aging. Patients with PD-CI showed greater GM-PAD (7.08 ± 6.64 years) and WM-PAD (8.82 ± 7.69 years) than those with PD-NCI (GM: 1.97 ± 7.13, *P*_adjusted_ = 0.011; WM: 4.87 ± 7.88, *P*_adjusted_ = 0.049) and controls (GM: -0.58 ± 7.04, *P*_adjusted_ = 0.004; WM: 0.88 ± 7.45, *P*_adjusted_ = 0.002) after adjusting demographic factors. In patients with PD, GM-PAD was negatively correlated with MMSE (*P*_adjusted_ = 0.011) and MoCA (*P*_adjusted_ = 0.013) and positively correlated with UPDRS Part II (*P*_adjusted_ = 0.036). WM-PAD was negatively correlated with logical memory of immediate and delayed recalls (*P*_adjusted_ = 0.003 and *P*_adjusted_ < 0.001). Also, altered brain regions in PD-CI were identified and significantly correlated with brain age measures, implicating the neuroanatomical underpinning of neurodegeneration in PD-CI. Moreover, the brain age metrics can improve the classification between PD-CI and PD-NCI. The findings suggest that patients with PD-CI had advanced brain aging that was associated with poor cognitive functions. The identified neuroimaging features and brain age measures can serve as potential biomarkers of PD-CI.

## Introduction

Parkinson’s disease (PD) is a neurodegenerative disease characterized by pathological alpha-synuclein aggregation, dopaminergic neuron loss in the substantia nigra pars compacta (SNpc), and motor symptoms such as rest tremor, bradykinesia, and gait disturbance^1^. In the advanced stage, alpha-synucleinopathy may affect cortices^2^, and most patients will experience cognitive impairment (PD-CI)^3^, which indicates a worse prognosis and affects the quality of life^4^. PD-CI is subcategorized as PD with mild cognitive impairment (PD-MCI)^5^ and PD with dementia (PDD)^6^. To achieve high clinical specificity and sensitivity, it is required to incorporate indispensable neuropsychological tests (NPT) into level II evaluations in conjunction with the utilization of the Montreal Cognitive Assessment (MoCA) for level I assessment. Nevertheless, these examinations necessitate considerable temporal investments and demand substantial manpower resources^7^. The dependability and explication of NPT outcomes may exhibit arbitrary discrepancies amongst raters and patients’ levels of cognizance. This variability may impede the early detection of the pathological conversion leading to PD-CI, for which no therapeutics are currently available for reversing its clinical progression. Therefore, an unmet clinical need exists for a robust and objective biomarker that can facilitate the detection of degenerative and senescent processes associated with PD-CI.

Brain magnetic resonance imaging (MRI) is acknowledged as a potential tool to provide objective, qualitative, and quantitative biomarkers. Brain age prediction has emerged as a new method for predicting brain health^8^. It entails predicting the brain age of individuals through machine learning algorithms and neuroimaging data^9–11^. The difference between the predicted brain age and chronological age is the predicted age difference (PAD), which has been suggested to be positively associated with the risk of various brain disorders such as Alzheimer’s disease^8,12,13^. Numerous investigations have demonstrated that increased PAD serves as a risk factor for cognitive impairment^14–16^; however, whether PD with varying degrees of cognitive impairment exhibited deviant trajectories of brain aging relative to healthy controls (HCs) has remained uncertain in prior studies^16–19^.

**Fig. 1:**
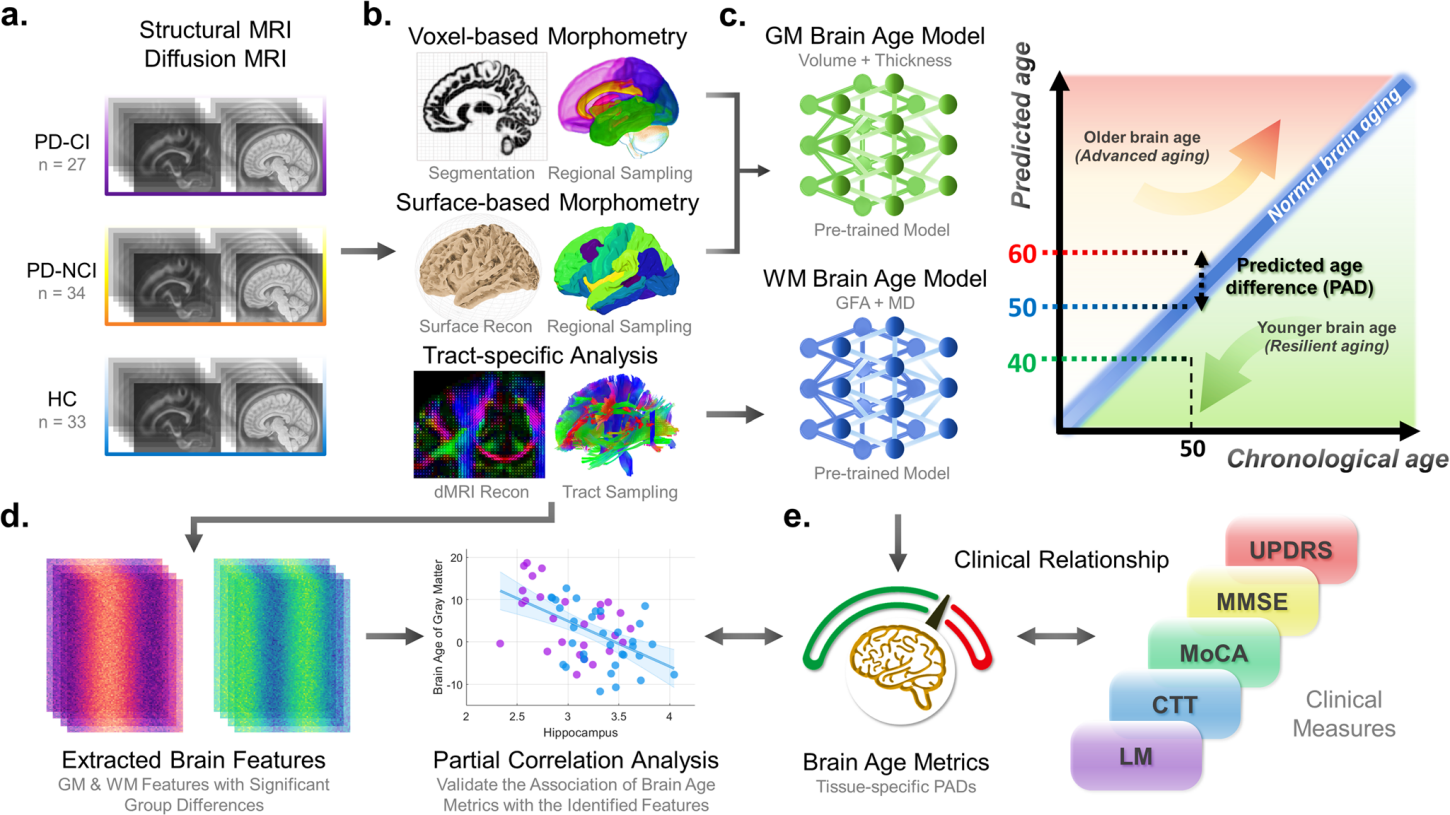
Flowchart of image processing, brain age estimation, and data analysis. Structural and diffusion MRI data were obtained for each group (**a**). The structural MRI data were analyzed using voxel-based and surface-based morphometries to extract volumetric and cortical thickness measures of gray matter (GM), respectively (**b**). Also, the diffusion MRI data were processed with tract-based analysis to extract white matter (WM) features (**b**). These imaging features from each modality were utilized to estimate tissue-specific brain age measures (**c**). Subsequently, the GM and WM brain-predicted age difference (PAD) metrics, representing the degree of brain aging, were used for further analyses. Brain features exhibiting significant between-group differences were extracted to examine their association with brain age measures, decomposing the potential contributing regions to advanced brain aging (**d**). Moreover, the clinical significance of brain age metrics was examined by analyzing their association with clinical measures of symptom severity and cognitive impairment (**e**).

To investigate the association of brain age with PD-CI, we employed two brain age metrics (i.e. PAD) derived from gray matter (GM)- and white matter (WM)-based neuroimaging features to quantify the degree of brain aging in patients with PD-CI, patients with PD but without substantially cognitive impairment (i.e. PD-NCI), and HCs (Fig. 1). Significantly greater PAD metrics represent older-appearing brain aging status relative to individuals’ chronological age. We also examined the associations of PAD measures with multiple clinical factors (e.g. symptom severity and cognitive scores). We hypothesized that patients with PD-CI would exhibit a greater PAD compared to patients with PD-NCI and HCs, and that PAD would be correlated with cognitive scores. Moreover, we attempted to identify specific neuroimaging features that could contribute to the advanced brain age in patients with PD-CI relative to PD-NCI and HC. Lastly, we assessed the clinical potential of neuroimaging-derived PAD metrics by performing classification between three experimental cohorts.

## Results

### Characteristics of participants

Patients with PD-CI (*n*, 27; mean age, 75.3 years; standard deviation (SD), 7.2; sex, 15 men), PD-NCI (*n*, 34; mean age, 70.0 years; SD, 7.9; sex, 20 men), and 33 HCs (mean age, 65.2 years; SD, 5.6; sex, 17 men) were recruited in this study. Patients with PD-CI consisted of 15 patients with PD-MCI (mean age, 74.1 years; SD, 7.8; sex, 6 men) and 12 patients with PD-D (mean age, 76.8 years; SD, 6.4; sex, 9 men). The details of recruitment criteria and clinical diagnosis are shown in Methods Participants. As presented in Table 1, the patients with PD-CI were chronologically older than those with PD-NCI and the HCs, so age was adjusted in the following analyses. The two PD groups did not differ significantly in sex or education level. Disease duration (*P* = 0.116) and onset age (*P* = 0.181) were slightly greater in the PD-CI group than in the PD-NCI group, but this difference was nonsignificant. Regarding symptom severity, the PD-CI group had significantly higher scores on the Hoehn and Yahr (H&Y) scale and Unified Parkinson’s Disease Rating Scale (UPDRS) (Part II, Part III, and overall score) than did the PD-NCI group (*P* < 0.001 for all items). In routine cognitive screening (RCS) examinations (i.e. Mini-Mental State Examination [MMSE] and MoCA scores), the PD-CI group had significantly lower scores than did the PD-NCI and HC groups, and the PD-NCI group had significantly lower scores than did the HC group; these results were all adjusted for age and education level (*P* < 0.001 for all items). Besides, NPTs including the Color Trails Test (CTT)^20^ for executive function and Wechsler Memory Scale (WMS)^21^ for logical memory (LM) were assessed; the PD-CI exhibited significantly longer response time in CTT and poorer memory performance in WMS compared to the PD-NCI (adjusted for age and education, *P* < 0.01 for all items, except WMS-LM_delayed_ *P* = 0.011).

**Table 1:** Characteristics of participants

Characteristic	PD-CI	PD-NCI	HC	*P*-value
*N*	27	34	33	*-*
Age (y)	75.3 (7.2)	70.0 (7.9)	65.2 (5.6)	<0.001 (PD-CI vs. PD-NCI: 0.008) (PD-CI vs. HC: <0.001) (PD-NCI vs. HC: 0.006)
Male (%)	55.6	58.8	51.5	0.834
Education (y)	9.8 (4.9)	11.1 (4.8)	15.1 (4.1)	<0.001 (PD-CI vs. PD-NCI: 0.326) (PD-CI vs. HC: <0.001) (PD-NCI vs. HC: 0.001)
MMSE	23.4 (4.3)	27.6 (1.7)	29.4 (0.8)	<0.001 (PD-CI vs. PD-NCI: <0.001) (PD-CI vs. HC: <0.001) (PD-NCI vs. HC: <0.001)
MoCA	17.7 (6.2)	25.3 (3.8)	28.4 (1.8)	<0.001 (PD-CI vs. PD-NCI: <0.001) (PD-CI vs. HC: <0.001) (PD-NCI vs. HC: <0.001)
Age at onset (y)	69.2 (8.3)	65.9 (8.8)	–	0.181
Disease duration (y)	6.2 (3.2)	4.8 (3.1)	–	0.116
Hoehn & Yahr scale	2.5 (0.9)	1.7 (0.7)	–	<0.001
UPDRS I	3.2 (2.3)	2.4 (1.7)	–	0.100
UPDRS II	11.3 (6.8)	5.4 (4.2)	–	<0.001
UPDRS III	15.8 (10.2)	9.6 (5.8)	–	0.004
UPDRS total	30.3 (15.3)	17.4 (9.7)	–	<0.001
CTT-1 RT	175.0 (97.3)	65.5 (36.6)	–	0.004
CTT-2 RT	291.5 (138.8)	132.0 (66.8)	–	0.001
WMS-LM_immediate_	15.7 (5.4)	25.5 (6.1)	–	<0.001
WMS-LM_delayed_	3.0 (2.0)	5.4 (3.0)	–	0.011

Note: mean (SD).

*PD-CI* Parkinson’s disease with cognitive impairment, *PD-NCI* Parkinson’s disease without cognitive impairment, *HC* healthy control, *MMSE* mini-mental state examination, *MoCA* Montreal cognitive assessment, *UPDRS* Unified Parkinson’s Disease Rating Scale, *CTT* Color Trails Test, *RT* response time, *WMS* Wechsler Memory Scale, *LM* logical memory.

### Comparison of PAD among PD-CI, PD-NCI, and HC groups

**Fig. 2:**
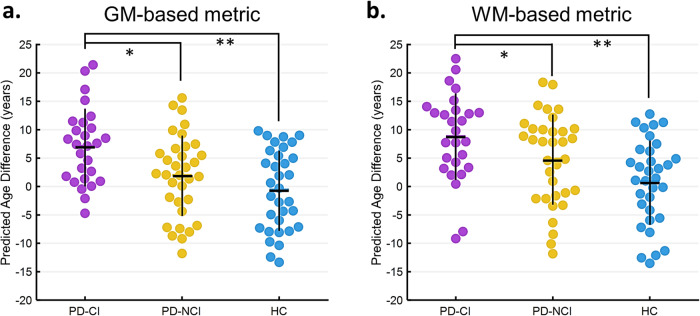
Comparison of PAD measures across groups. The PAD metrics derived from GM (**a**) and WM (**b**) features are visualized with beeswarm plots. Vertical and horizontal lines that overlap observation dots indicate interquartile range and median, respectively. Asterisk sign * and ** indicate adjusted *P* < 0.05 and adjusted *P* < 0.01, respectively. The PAD measures displayed were adjusted by ANCOVA given the mean values of continuous covariates (i.e. age and education) and female class of the sex covariate. GM gray matter, WM white matter, PD Parkinson’s disease, CI cognitive impairment, NCI non-cognitive impairment, HC healthy control.

The GM- and WM-PAD measures were compared among the PD-CI, PD-NCI, and HC groups using analyses of covariance (ANCOVAs) adjusted by chronological age, sex, and education, and the *post hoc* analysis examined between-group differences with the Benjamini–Hochberg method for multiple comparison adjustment. The GM-PADs of the three groups differed significantly (PD-CI, 7.08 ± 6.64 years; PD-NCI, 1.97 ± 7.13 years; HC, −0.58 ± 7.04 years; *F*_(2,88)_ = 6.25, adjusted *P* = 0.006; Fig. 2a). Notably, the mean PAD measures displayed were the estimated marginal means adjusted by ANCOVA given the mean values of continuous covariates and female class of the sex covariate. The subsequent *post hoc* analysis achieved by ANCOVA adjusting for the same covariates revealed significantly advanced GM brain aging in the PD-CI group relative to the HC group (PD-CI vs. HC, adjusted *P* = 0.004) and PD-NCI group (PD-CI vs. PD-NCI, adjusted *P* = 0.011), but no significant difference was observed between the PD-NCI and HC groups (adjusted *P* = 0.353). Regarding WM-PAD, the three groups differed significantly (PD-CI, 8.82 ± 7.69 years; PD-NCI, 4.87 ± 7.88 years; HC, 0.88 ± 7.45 years; *F*_(2,88)_ = 5.24, adjusted *P* = 0.007; Fig. 2b). *Post hoc* analysis with the same covariate adjustments revealed significantly greater WM aging in the PD-CI group relative to the HC group (PD-CI vs. HC, adjusted *P* = 0.002) and PD-NCI group (PD-CI vs. PD-NCI, adjusted *P* = 0.049); however, no significant difference was detected between the PD-NCI and HC groups (adjusted *P* = 0.447). To further elucidate the potential influence of motor-specific symptoms on the observed disparity in PAD metrics between PD-CI and PD-NCI, a subgroup comparison was conducted with the inclusion of an additional covariate, the UPDRS part III (motor-specific symptom severity). The results showed that the GM-PAD was significantly greater in PD-CI compared to PD-NCI (adjusted *P* = 0.027) while the difference in WM-PAD did not reach statistical significance (adjusted *P* = 0.114) (Supplementary Note 1). This suggested that WM-PAD was more susceptible to the combined impact of both cognitive and motor impairment relative to GM-PAD.

### Association of PAD with clinical variables

**Fig. 3:**
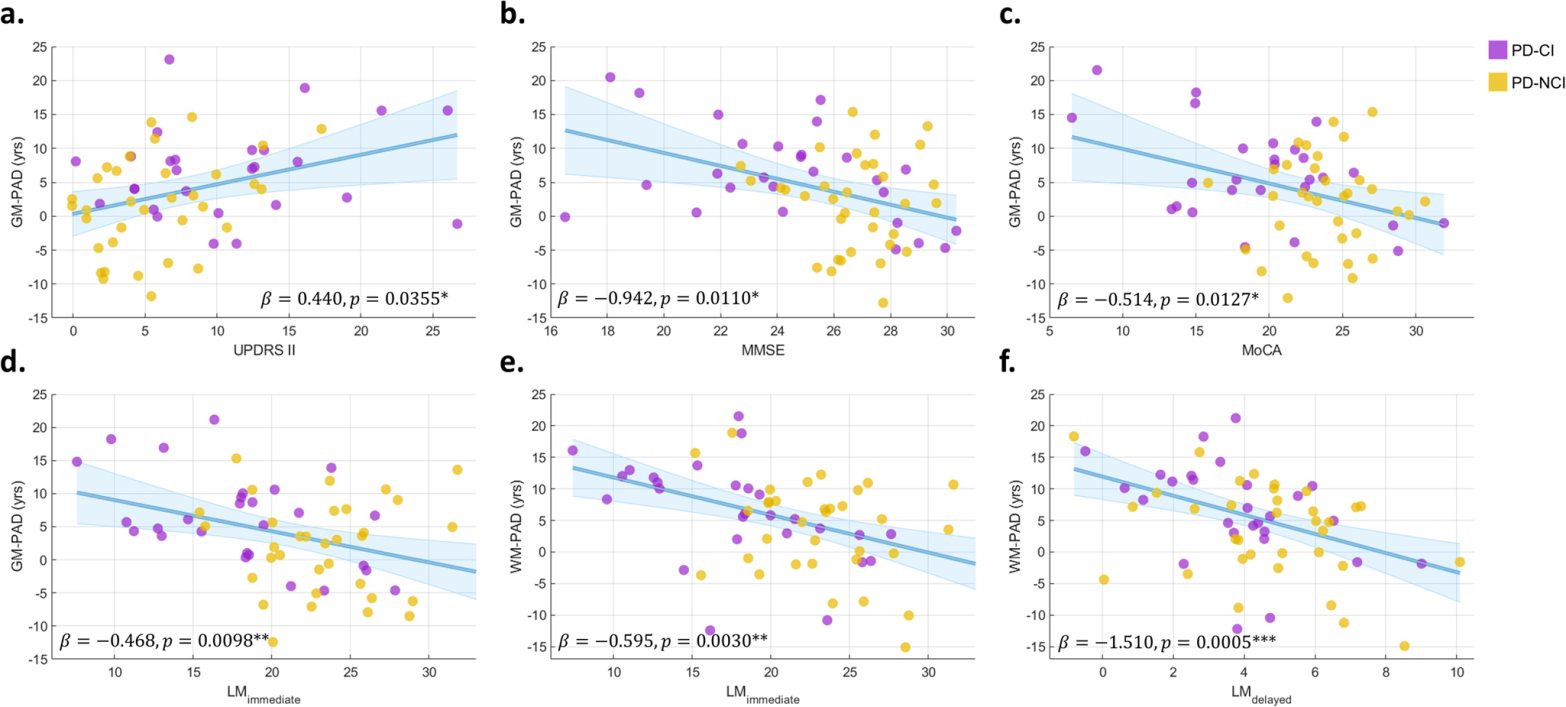
Associations of PAD measures with symptom severity and cognitive measures. Subplots (**a**–**c**) show statistical association of GM-PAD metric with UPDRS part II score, MMSE, and MoCA, respectively. Subplots (**d**–**f**) demonstrate statistical association of LM measures with GM-PAD and WM-PAD, respectively. Patients with and without cognitive impairment are illustrated with purple and yellow dots, respectively. Presented variables are adjusted for covariates shown in Methods Statistical Analysis. Significance level: * adjusted *P* < 0.05, ** adjusted *P* < 0.01, *** adjusted *P* < 0.001. UPDRS Unified Parkinson’s Disease Rating Scale, MoCA Montreal Cognitive Assessment, MMSE Mini-Mental State Examination, LM logical memory, GM gray matter, WM white matter, PD Parkinson’s disease, CI cognitive impairment.

Multiple linear regression was used to examine the influence of clinical factors on PAD measures; the regression analysis included five categories of clinical dimensions: symptom severity, time-related clinical factors, routine cognitive screening outcomes, executive function, and logical memory. Table 2 lists the associations of PAD with multiple clinical variables including symptom severity, illness duration, onset age, general cognitive performance, and domain-specific cognitive performance, in patients with PD. We observed significant associations of brain age metrics with symptom severity, general cognitive outcomes, and logic memory-specific performance (Fig. 3) but not in the time-related clinical factors (i.e. onset age and duration of illness) and executive function. The symptom related to experiences of daily living (assessed using the UPDRS part II) was significantly associated with the increased GM-PAD (β = 0.440, adjusted *P* = 0.036) and showed a trend with the increased WM-PAD (β = 0.416, adjusted *P* = 0.092). The disease severity, as assessed using the total UPDRS score, also revealed a statistical trend with the increased GM-PAD (β = 0.166, adjusted *P* = 0.080). The onset age tended to be marginally associated with the decreased GM-PAD (β = −0.252, adjusted *P* = 0.052). The GM-PAD but not WM-PAD was significantly and negatively associated with MMSE (β = −0.942, adjusted *P* = 0.011) and MoCA (β = −0.514, adjusted *P* = 0.013) scores. The CTT measures did not show significant association with PAD metrics; however, the WMS-LM immediate score was significantly and negatively associated with both PAD metrics (β = −0.468, adjusted *P* = 0.010 for GM-PAD and β = −0.595, adjusted *P* = 0.003 for WM-PAD). Also, the WMS-LM delayed score was significantly correlated with the decreased WM-PAD (β = −1.510, adjusted *P* < 0.001) and reflected a trend with the decreased GM-PAD (β = −0.795, adjusted *P* = 0.065).

**Table 2:** Associations of PAD with symptom severity, cognitive measures, and other clinical factors

	GM-PAD			WM-PAD		
	Estimate	SE	*P*-value	Estimate	SE	*P*-value
UPDRS I	0.804	0.500	0.2270	0.999	0.496	0.1461
UPDRS II	**0.440**	**0.158**	0.0355^*^	0.416	0.171	0.0915^†^
UPDRS III	0.134	0.130	0.3061	0.192	0.125	0.2624
UPDRS total	0.166	0.069	0.0799^†^	0.175	0.077	0.1068
H&Y scale	2.172	1.106	0.1644	0.207	1.248	0.8689
Duration of illness	0.604	0.335	0.1542	0.424	0.382	0.2726
Onset age	−0.252	0.126	0.0518^†^	−0.215	0.133	0.2214
MMSE	**−0.942**	**0.326**	0.0110^*^	−0.502	0.340	0.1460
MoCA	**−0.514**	**0.200**	0.0127^*^	−0.368	0.199	0.1382
CTT-1 RT	0.017	0.011	0.2224	0.015	0.011	0.3240
CTT-2 RT	0.009	0.011	0.3967	0.014	0.011	0.2012
LM_immediate_	**−0.468**	**0.160**	0.0098^**^	**−0.595**	**0.164**	0.0030^**^
LM_delayed_	−0.795	0.423	0.0654^†^	**−1.510**	**0.408**	0.0005^***^

Multiple linear regression was performed with Benjamini–Hochberg adjusted significance level.

*PAD* predicted age difference, *GM* gray matter, *WM* white matter, *UPDRS* Unified Parkinson’s Disease Rating Scale, *H&Y* Hoehn & Yahr Scale, *MMSE* Mini-Mental State Examination, *MoCA* Montreal Cognitive Assessment, *CTT* Color Trails Test, *R*T response time, *LM* logical memory, *SE* standard error.

^†^Adjusted *p*-values < 0.1.

*Adjusted *p*-values < 0.05.

**Adjusted *p*-values < 0.01.

***Adjusted *p*-values < 0.001.

### Contributions of features to advanced brain age in PD-CI

**Fig. 4:**
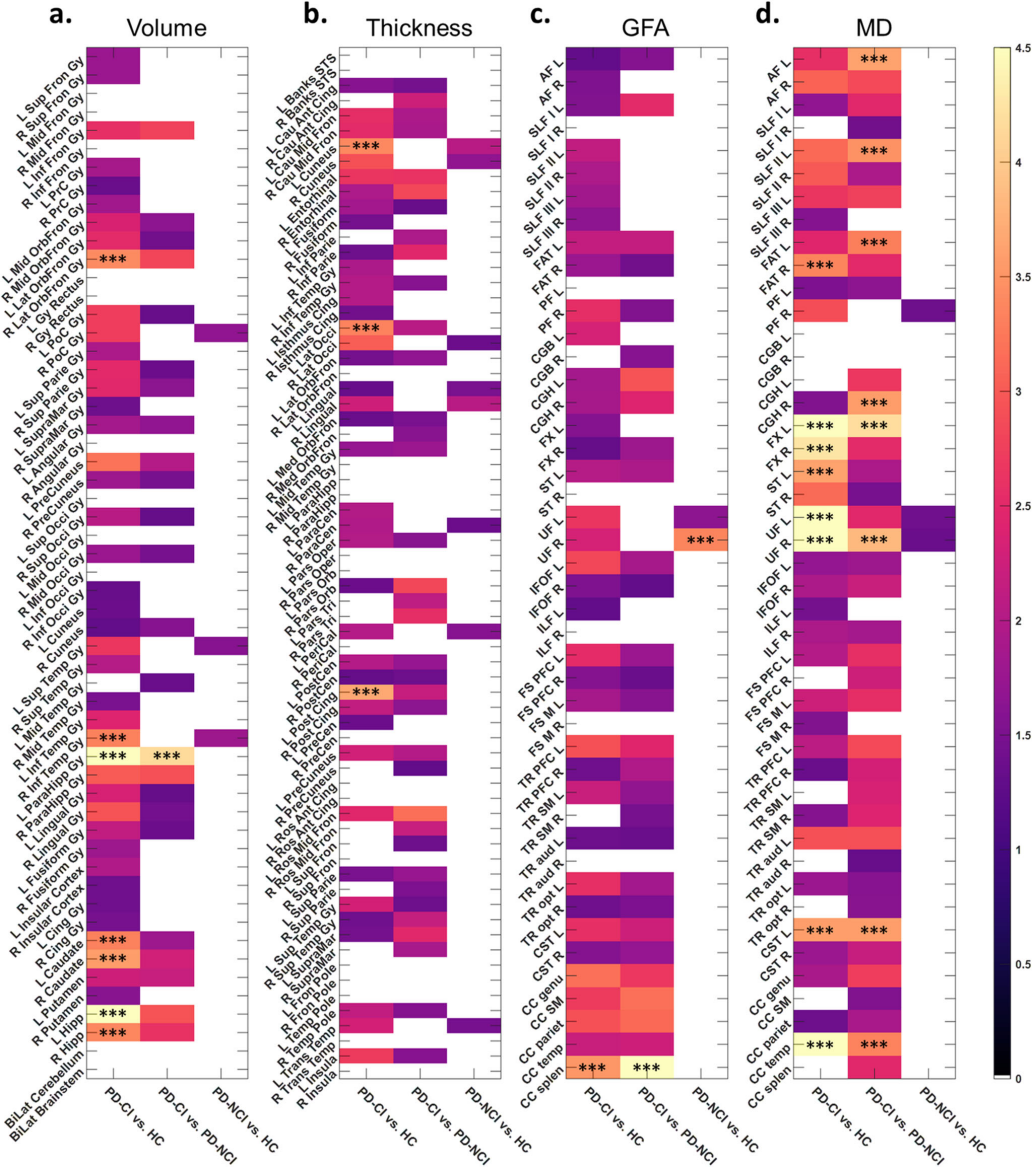
Potential key features of gray matter (GM) and white matter (WM) contributing to the discrepancy between groups for advanced brain age. Values in the density plot for GM volume (**a**), GM thickness (**b**), WM GFA (**c**), and WM MD (**d**) indicate a negative logarithm to base 10 of *P* values corresponding to group differences; higher values indicate greater significance (corresponding brighter ones in the color scale). Anatomical regions with *P*-values less than 0.05 are visualized, and those with *P*-values less than 0.001 are labeled with asterisks. Full names of anatomical regions are provided in Supplementary Note 4. GFA generalized fractional anisotropy, MD mean diffusivity.

To identify the image features attributable to advanced brain aging in PD-CI, we first tested for image feature differences between the PD-CI and HC groups (see Methods Statistical Analysis). The comparisons of image features through mass univariate ANCOVAs (adjusting age, sex, and education) revealed that, relative to the HC group, the PD-CI group had 44 GM features with substantially (*P* < 0.05) lower values for volumetric measures and 40 GM features with substantially less cortical thickness (Fig. 4a, b and Supplementary Table 1). Left and right side features were calculated separately. Within them, volume in the right lateral orbitofrontal gyrus, right inferior temporal gyrus, left parahippocampal gyrus, bilateral caudate, and bilateral hippocampi (Fig. 4a) and cortical thickness in the left cuneus, left lateral occipital gyrus, and left posterior cingulate gyrus (Fig. 4b) were considered as the significantly distinct features (*P* < 0.001). For WM features, the PD-CI group had 35 substantially higher mean diffusivity (MD) values and 37 substantially lower generalized fractional anisotropy (GFA) values relative to the HC group especially for those significant features such as the MD in the right frontal aslant tract, left stria terminalis, bilateral uncinate fasciculi, bilateral fornices, left cerebrospinal tract, and the corpus callosum of the temporal lobes and the GFA in the corpus callosum of the splenium (Fig. 4c, d and Supplementary Table 2). Moreover, we identified convergence between the aforementioned differentiated features in PD-CI compared to HC and those demonstrating statistically significant differences (*P* < 0.001) between PD-CI and PD-NCI. This overlap was evident in regions including the volume of the left parahippocampal gyrus, the MD of the left fornix, right uncinate fasciculus, left cerebrospinal tract, and the corpus callosum of temporal lobes, and GFA of the corpus callosum of the splenium, suggesting that these features may also serve as potential differential markers for PD-CI and PD-NCI.

**Fig. 5:**
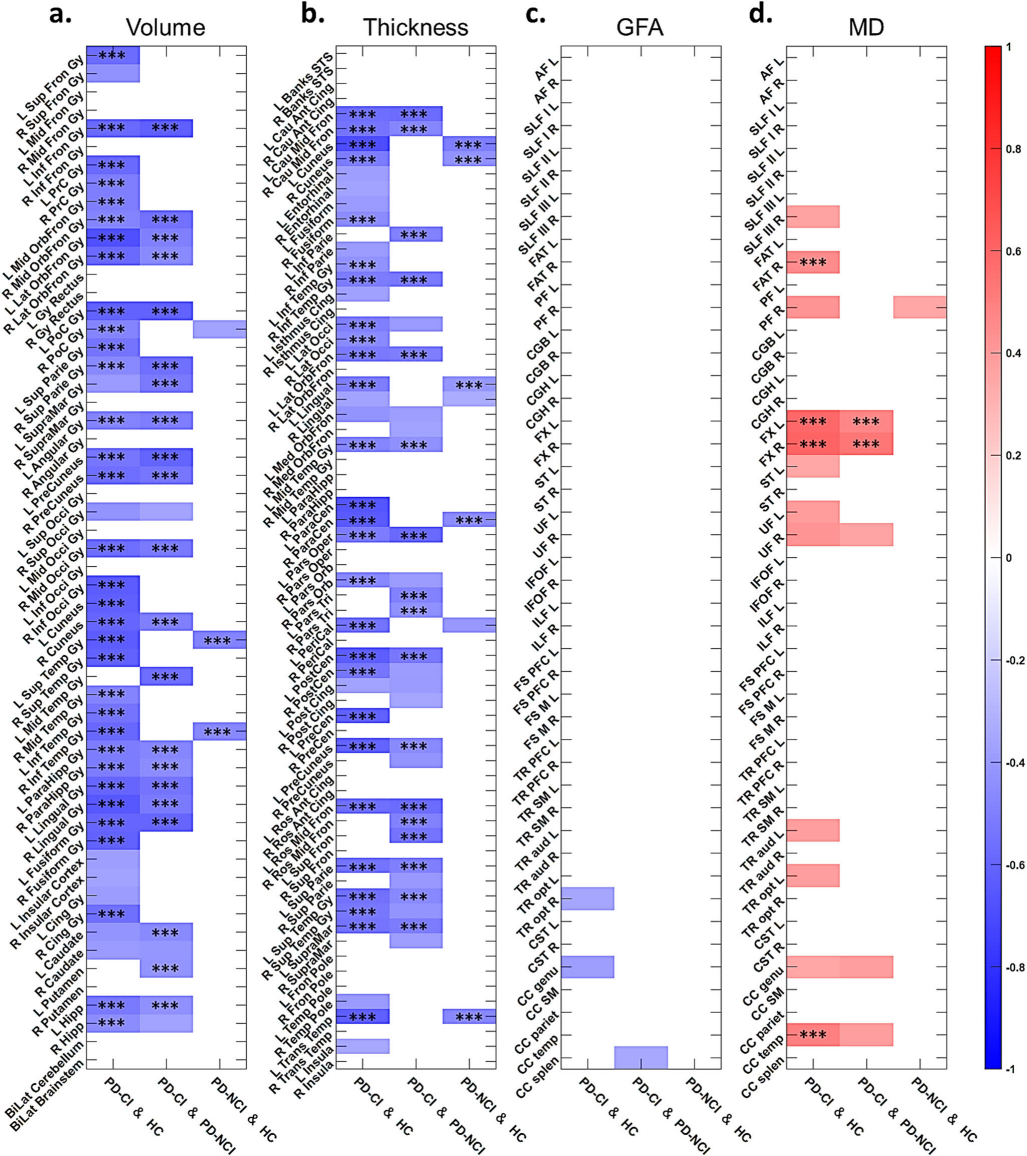
Partial correlation analysis identifying the association of image features with brain age metrics. Values in the density plot for volume (**a**), thickness (**b**), GFA (**c**), and MD (**d**) indicate linear correlations of individual image features with brain age metrics while adjusting age, sex, and education. Red and blue color spectrums indicate positive and negative correlation, respectively. Anatomical regions with *P*-values less than 0.01 are visualized, and those with *P*-values less than 0.001 are labeled with asterisks. Full names of anatomical regions are provided in Supplementary Note 4. GFA generalized fractional anisotropy, MD mean diffusivity.

To further examine the role of highlighted image features in the advanced brain aging in PD-CI as compared with HC, partial correlation analysis was applied to the selected features (*P* < 0.05, listed in Fig. 4) within each tissue type (GM and WM) while adjusting age, sex, and education to assess their potential contributions to brain age metrics. The results revealed that the majority of GM regions demonstrating substantial differences between PD-CI and HC were also negatively correlated with their GM-PAD (PD-CI & HC) such as the right lateral orbitofrontal gyrus, right inferior temporal gyrus, bilateral parahippocampal gyri, and bilateral hippocampal volumes (Fig. 5a) as well as the bilateral cuneus, bilateral lateral occipital gyri, and bilateral caudal middle frontal cortical thickness measures (Fig. 5b). In contrast, although numerous WM metrics substantially differed between PD-CI and HC, only a limited fraction of WM bundles exhibited significant associations with their WM-PAD including the MD in the right frontal aslant tract, bilateral fornices, and the corpus callosum of the temporal lobes (Fig. 5d). The resulting features not only reflects the impaired brain structures in patients with PD-CI but were also substantially associated the variation in brain age between the PD-CI and HC groups. This implied that the advanced GM aging in PD-CI may stem largely from widespread deterioration of GM structures whereas advanced WM aging in PD-CI may originate from localized impairments in specific WM tracts.

Similarly, in the analysis for PD-CI and PD-NCI, most of the differential features in GM were significantly negatively associated with their GM-PAD (PD-CI & PD-NCI) including the bilateral lateral orbitofrontal gyri, bilateral parahippocampal gyri, and left hippocampus in volume (Fig. 5a) as well as the bilateral caudal middle frontal gyri and bilateral pars triangularis in thickness measures (Fig. 5b). In the WM features, only bilateral fornices showed significantly positive correlation with their WM-PAD (Fig. 5d). These findings suggest that the identified features might explain the association with brain age between the PD-CI and PD-NCI, highlighting the potential neuroanatomical underpinning of cognitive impairment. The results from comparing the PD-NCI and HC groups are provided as a reference.

### Improvement of classification between PD-CI and PD-NCI by using brain age metrics

**Fig. 6:**
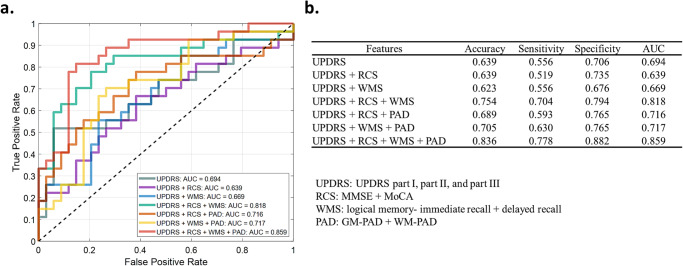
Receiver operating characteristic (ROC) analysis for PD-CI classification based on clinical factors and brain age metrics. The ROC curves (**a**) were yielded by using different combinations of clinical factors and/or brain age metrics. The performance was evaluated by using 5-fold cross-validation (**b**). AUC Area Under ROC Curve, PAD Predicted Age Difference, GM Gray Matter, WM White Matter, UPDRS Unified Parkinson’s Disease Rating Scale, MMSE Mini-Mental State Examination, MoCA Montreal Cognitive Assessment, WMS Wechsler Memory Scale.

In order to evaluate the clinical impact of brain age metrics as a diagnostic-aided marker, we conducted a classification task to differentiate PD-CI and PD-NCI groups by using different combinations of clinical factors (UPDRS) as well as brain age metrics and assessed the performance by using receiver operating characteristic (ROC)^12^ analysis. By using the support vector machine with a cubic kernel through 5-fold cross-validation, the baseline model using UPDRS achieved an accuracy of 63.9% and area under the ROC curve (AUC) = 0.694 (Fig. 6b). By solely adding routine cognitive screening (RCS) scores (i.e. MMSE and MoCA) or WMS scores into the model (CTT was excluded due to lack of statistical relevance), the performance cannot be improved (Fig. 6); however, combining these two dimensions can improve performance metrics (accuracy 75.4% and AUC = 0.818). Moreover, the classification performance can be widely improved by including brain age metrics (Fig. 6b). With brain age metrics as an additional predictor, the accuracy, sensitivity, and specificity can be averagely increased by 10.6%, 12.7%, and 9.4%, respectively. The overall model combining all selected clinical factors and brain age metrics can achieve an accuracy of 83.6% and AUC = 0.859, which increased by 19.7% compared to the baseline model. This result suggested that the brain age metrics could provide additional information to facilitate the differentiation between PD-CI and PD-NCI.

## Discussion

Using brain age metrics, we demonstrated that both GM-PAD and WM-PAD in patients with PD-CI were significantly increased (approximately +7–8 years) compared with those in patients with PD-NCI (approximately +2–4 years) and HCs (approximately zero). GM- and WM-PAD were both negatively associated with immediate recall in logical memory. WM-PAD was negatively associated with delayed recall of logical memory. Only GM-PAD was negatively correlated with routine cognitive screening (MMSE and MoCA) and positively associated with impaired activities of daily living (UPDRS Part II). This supports the clinical validity of brain age metrics in GM and WM for assessing cognitive alterations in PD^16,19^. Previous literature suggests AD patterns of brain atrophy may be a preclinical biomarker of cognitive decline in PD^22^. In our study, the elevated PAD in the PD-CI group was largely attributed to regional GM and WM features that bore a resemblance to the characteristics of AD such as parahippocampal regions (Fig. 4). Through this paradigm, the diagnostic accuracy of PD-CI was improved by using brain age metrics (Fig. 6). These findings suggest the relevance of the image marker in the early detection of PD-CI.

A more extensive and advanced GM aging was clearly observed in PD-CI compared to PD-NCI. The volumetric measures including the bilateral hippocampi, bilateral parahippocampi, right inferior temporal gyrus, and right lateral orbitofrontal gyrus showed a significant association with GM-PAD when there was lower cognitive performance. These findings may correlate to autopsied studies of alpha-synuclein^23^ and other co-pathology of amyloid-beta and tau in PD-CI^24^. Our results observed AD-associated GM patterns in PD, specifically those with cognitive impairment, in terms of advanced GM-PAD, which was consistent with previous studies^16,19,22^. This may suggest the involvement of the aberrant basal forebrain cholinergic system in PD-CI^25^. Additional positron emission tomography imaging is warranted to clarify whether advanced GM-PAD would be correlated with amyloid or tau positivity^16^. Other brain areas related to advanced brain age (e.g. lateral orbitofrontal lobes, cuneus, lateral occipital gyri, parahippocampi, and hippocampi) have been demonstrated to have functional effects on cognition in patients with PD^26,27^. For example, the circuit involving the orbitofrontal lobes to the ventral caudate nucleus and that from the dorsolateral prefrontal cortex to the dorsal caudate nucleus are two of the three key loops in the pathogenesis of frontostriatal cognitive dysfunction in patients with PD^28^. Similarly, the reduced thickness of the left cuneus and left lateral occipital gyrus may functionally underlie the common presentation of visual hallucination in patients with PD^29^, and this condition is substantially worsened after the development of cognitive impairment. On the basis of these findings, these GM areas warrant further research to clarify their functional effect on motor and nonmotor symptoms in patients with PD-CI.

In our analysis using WM-PAD, we revealed a higher degree of involvement of impaired WM tracts in the PD-CI group compared to both the PD-NCI and HC groups. These identified WM features, particularly including the fornices, corpus callosum, uncinate fasciculi, frontal aslant tracts, and superior longitudinal fasciculi, play a key role in aberrant brain aging in patients with PD-CI. For example, the fornix is considered a major outflow hub for transducing signals from the hippocampus^30,31^. The MD of the fornix have been reported to predict cognitive impairment in patients with MCI and AD^30^ and to be correlated with memory function in patients with PD^32^. Additionally, WM abnormalities in the corpus callosum may contribute to PD-CI by disrupting information transfer across interhemispheric and callosal–cortical projections^33^. WM alterations were previously detected in multiple tracts such as the uncinate fasciculi, superior longitudinal fasciculi, and corpus callosum in patients with PD-MCI^34^. Considering that a higher WM-PAD was observed in PD-CI, the aforementioned aberrant WM alterations may impede the connections among various brain areas and impair cognitive domains that are not commonly identified in the current cognitive domains shown in AD spectrum diseases^14^. The association of WM-PAD with various high-level cognitive domains should be studied to clarify the clinical role of WM-PAD in PD-CI.

Our study demonstrated the advantage of using both GM-specific and WM-specific brain age metrics for investigating brain alterations in PD-CI. Nevertheless, it has several limitations. First, the sample size of our single-center cohort is limited, which may compromise our analysis of the associations between brain age and clinical variables. Therefore, future studies should include a larger validation cohort for the differentiation and prognosis of PD-CI. Second, we aggregated PD-MCI and PDD into a single PD-CI group, neglecting the differentiation of the two disease categories. However, the status of cognitive decline can be considered as reflecting a continuum of the disease spectrum instead of two distinct disease categories. Future studies can validate this concept by conducting a longitudinal assessment using the current cohort as a baseline. Third, predicted brain age may vary depending on the in-house analytic method and machine learning methods used. Nevertheless, we have minimized this concern by validating and implementing our analytic pipeline in several studies^9,10,18^. In addition, there were statistically significant differences in age and education between the clinical and control groups. To reduce the potential confounding influence of these variables, we utilized bias-free brain age metrics and adjusted for these covariates in all statistical analyses. Nevertheless, age-matched study design is warranted to control for aging-related pathological phenomena such as amyloid or tau positivity.

Using modality-specific brain age metrics, we identified the advanced brain aging in patients with PD-CI relative to patients with PD-NCI and HCs. This elevated brain aging can be attributed to the unique involvement of GM and WM areas characteristic of cognitive impairment. Moreover, we demonstrated clinical associations of brain age metrics with symptom severity, general cognitive performance, and memory-specific outcomes. Our findings suggest that the unique anatomical patterns of PD-CI can potentially serve as a diagnostic- or prognostic-aided imaging biomarker for PD-CI, paving the way for future advancements in early detection and personalized management of PD-CI. Further investigations should focus on conducting longitudinal assessments to validate the practicability of the identified distinctive patterns in enabling early identification of cognitive impairment in patients with PD.

## Methods

### Participants

Patients with PD-CI (*n*, 27; mean age, 75.3 years; standard deviation (SD), 7.2; sex, 15 men) and PD-NCI (*n*, 34; mean age, 70.0 years; SD, 7.9; sex, 20 men) were recruited from the outpatient clinic of the Department of Neurology, National Taiwan University Hospital (NTUH) between December 2019 and December 2020. Clinical diagnoses of PD were established using the criteria stipulated by the UK Brain Bank^35^ and were confirmed through Tc^99m^-TRODAT imaging performed by experienced neurologists from the PD center of NTUH. Patients with similar education levels and disease durations were preferably included whereas those with malignancies, autoimmune disorders, cerebrovascular disorders, major systemic diseases, self-reported substance abuse, brain surgery, or other known neuropsychiatric diseases were excluded. The patients’ symptoms at initial recruitment were assessed using the Unified Parkinson’s Disease Rating Scale (UPDRS) and H&Y scale. Cognitive function was first screened using the MoCA and MMSE. All patients underwent a complete NPT to confirm their cognitive impairment status including the Wechsler Memory Scale (WMS) and Color Trails Test (CTT); the former was used to assess memory-related deficits, and the latter evaluated cognitive processing speed, attention, and executive function. A total of 33 HCs (mean age, 65.2 years; SD, 5.6; sex, 17 men) who met the following inclusion criteria were enrolled: having MMSE (≥25) and MoCA (≥26) and not having any self-reported substance abuse, brain injury, severe ongoing health problems, and a history of neurological diseases or psychiatric disorders. The Institutional Review Board of NTUH approved the study (No: 201904092RINC), and all participants provided written informed consent. The raw neuroimaging data acquired are not available due to confidentiality agreement of NTUH Research Ethics Committee. The secondary data are conditionally available upon request from the corresponding author.

### Brain image acquisition through MRI

All brain images used in this study were acquired using a 3-Tesla MRI scanner (Tim Trio; Siemens, Erlangen, Germany) with a 32-channel phased-array head coil. We collected T1-weighted images and diffusion spectrum imaging (DSI) data sets to estimate GM and WM features, respectively. T1-weighted imaging was performed using a three-dimensional (3D) magnetization-prepared rapid gradient-echo sequence with the following parameters: repetition time/echo time (TR/TE) = 2000/3 ms; flip angle = 9°; field of view (FOV) = 256 × 192 × 208 mm^3^; and isotropic spatial resolution = 1 mm^3^. DSI was performed using a pulsed-gradient spin-echo echo-planar imaging sequence with the following parameters: *b*_max_ = 4000 s/mm^2^; TR/TE = 9600/130 ms; slice thickness = 2.5 mm; FOV = 200 × 200 mm^2^; and in-plane spatial resolution = 2.5 × 2.5 mm^2^. The acquisition scheme comprised 102 diffusion-encoding gradients that corresponded to the Cartesian grids in the half-sphere of a 3D diffusion-encoding space and employed bipolar diffusion-encoding gradient design to minimize the eddy current artifact at the sequence level^18^. Each MRI scanning process involved T1-weighted imaging (~3 min) and DSI (~16 min).

### Image analysis

Before executing image analyses, we subjected all T1-weighted images and DSI data to quality assessments (see Supplementary Note 2). All the structural and diffusion MRI data sets used in the present study exhibited satisfactory image quality. To extract GM features from the T1-weighted images, voxel-based morphometry and surface-based morphometry were performed using the Computational Anatomy Toolbox^36^. Voxel-based morphometry was applied to estimate regional volume features in accordance with the LONI probabilistic brain atlas, which contains 56 regions of interest^37^. Besides, surface-based morphometry was employed to estimate cortical thickness^36^, and the thickness features were sampled using 68 cortical regions of interest defined by the Desikan–Killiany atlas^38^. A total of 56 volumetric and 68 cortical thickness features were used to conduct GM-based brain age estimation. Details regarding the image processing procedures are provided in Supplementary Note 3.

WM features were extracted from DSI data sets using an in-house analytic pipeline to convert DSI data into tract-specific features^11,39^. The algorithm applied is described in Supplementary Note 3, and the code of DSI imaging process is conditionally available upon request from the corresponding author. In brief, diffusion indices (i.e. GFA and MD) were reconstructed from DSI data by using the regularized mean apparent propagator MRI algorithm^40^. We sampled tract-specific features according to 45 predefined tract bundle coordinates. Consequently, 45 GFA and 45 MD features were obtained to execute WM-based brain age estimations. The parcellation of GM and WM is described in Supplementary Note 4.

### Brain age modeling and estimation

The pre-trained brain age models used in the present study were established and validated in our previous study^10,18,41^. In brief, GM-based and WM-based brain age prediction models were established using 124 GM features and 90 WM features from the training set, respectively, plus sex factor, which provided sex representation in the aging process. We employed a 12-layer feed-forward cascade neural network architecture for brain age prediction^41^. Since all images acquired in this study and the image dataset used to train the brain age models were from the same MRI scanner, feature harmonization was not employed before brain age prediction. To detect subtle differences between PD-CI and PD-NCI, we leveraged the concept of continual learning and executed domain adaptation on the brain age models^42^; this adaptation procedure enabled the models to provide a better and unbiased fit for the elderly population. The details are presented in Supplementary Note 5. After assessing the models’ performance, we estimated the bias-free GM-based and WM-based PAD scores^43^ for the PD-CI, PD-NCI, and HC groups for further analyses. The methodologies of brain age modeling and analytic scripts are available in our published work^41^.

### Neuropsychological assessment

Besides applying routine cognitive screening (i.e. MMSE & MoCA), all patients received the same neuropsychological test battery. Color Trails Tests with parts I and II (CTT I and II) were applied to assess patients’ executive function^20^. The response time of the task was recorded to reflect the cognitive performance of processing speed. Also, the logical memory (LM) was evaluated by Wechsler Memory Scales (WMS), third edition, in terms of immediate and delayed recalls^21^. The lower score indicates poorer LM outcomes.

### Statistical analysis

We compared the GM- and WM-PAD scores of the PD-CI, PD-NCI, and HC groups by performing analyses of covariance (ANCOVAs) adjusted for chronological age, sex, and education. A *post hoc* analysis achieved by ANCOVA was used to test between-group differences while adjusting age, sex, and education. Benjamini–Hochberg method was used to address the multiple-comparison problem in the initial multiple comparison tests of brain age metrics and the *post hoc* analyses.

We conducted univariate linear regression to investigate clinical factors influencing PAD measures. The clinical dimensions examined in the multiple linear regression analysis were divided into five categories: (1) symptom severity (total and subdivided scores for the UPDRS and H&Y scale), (2) time-related clinical factors (i.e. duration of illness and onset age), (3) routine cognitive screening outcomes (i.e. MMSE and MoCA scores), (4) executive function (i.e. response time in CTT), and (5) logical memory (i.e. WMS-LM immediate and delayed outcomes). Models corresponding to (1) symptom severity and (2) time-related clinical factors were analyzed using the covariates (1) age and sex and (2) sex, respectively. Models for routine cognitive screening and domain-specific cognitive examinations were adjusted for age, sex, education, and UPDRS motor-specific subset (part III). The analyses were performed for all patients with PD, and the Benjamini–Hochberg method was used to address the multiple-comparison problem within each dimension.

We further investigated the contribution of image features to the observed advanced brain aging in the PD-CI. This exploratory analysis involved two steps; first, mass univariate ANCOVAs were conducted to identify the differences in image features for each pair of the groups (i.e. PD-CI vs. HC, PD-CI vs. PD-NCI, and PD-NCI vs. HC) with the adjustment for age, sex, and education. We then selected features with significant between-group differences (uncorrected *P* < 0.05) as candidates. Second, partial correlation analysis was applied to the selected features under each tissue type (i.e. GM & WM) to test the relevance between image features and brain age metrics, which may represent individual linear contributions of image features to brain age estimates. In practice, each GM and WM feature was individually correlated with GM- and WM-PAD, respectively, while adjusting age, sex, and education (significance level as uncorrected *P* < 0.01). The features that exhibited both statistical between-group differences and statistical correlation with brain age metrics may potentially serve as neuroanatomical underpinning of aberrant brain aging in PD-CI.

Furthermore, to assess whether the brain age metrics are capable of serving as diagnostic-aided markers, we conducted a classification task to differentiate PD-CI and PD-NCI by using clinical factors as well as brain age metrics. The clinical factors covered those features demonstrating significant differences between these two groups including the UPDRS, RCS, and WMS. The classifiers were implemented by using support vector machine^44^ with a cubic kernel and evaluated by the ROC analysis and performance metrics including accuracy, sensitivity, and specificity through 5-fold cross-validation.

### Reporting summary

Further information on research design is available in the Nature Research Reporting Summary linked to this article.

### Supplementary information

Supplementary materials

Reporting summary

## Data Availability

The raw neuroimaging data acquired from the National Taiwan University Hospital (NTUH) are not available due to confidentiality agreement of the NTUH Research Ethics Committee. The secondary data are conditionally available upon request from the corresponding author. The methodologies of brain age modeling and analytic scripts are available in our published paper including the URL to the online open-access repository (Chen, C. L. et al. Generalization of diffusion magnetic resonance imaging-based brain age prediction model through transfer learning. Neuroimage 217, 116831, 10.1016/j.neuroimage.2020.116831 (2020)). The code of the imaging process is conditionally available upon request from the corresponding author.
