# Supplementary material for: Advanced brain aging in Parkinson’s disease with cognitive impairment
Source: NPJ Parkinsons Dis. 2024 Mar 16;10:62. doi: 10.1038/s41531-024-00673-7 (PMC10944471; doi:10.1038/s41531-024-00673-7)
Supplement: Supplementary file 1 — Supplementary materials [file 41531_2024_673_MOESM1_ESM.pdf]

Supplementary Materials

Supplementary Note 1: Subgroup Analysis of Brain Age Metrics in PD-CI and PD-NCI

Supplementary Note 2: Image Quality Assurance

Supplementary Note 3: Image Data Processing

Supplementary Note 4: Abbreviations for Anatomical Regions in Gray Matter and White Matter

Supplementary Note 5: Performance of Brain Age Prediction Models

Supplementary Table 1: Gray Matter Differences in PD-CI, PD-NCI, and HC

Supplementary Table 2: White Matter Differences in PD-CI, PD-NCI, and HC

Supplementary Note 1: Subgroup Analysis of Brain Age Metrics in PD-CI and PD-NCI

To better understand if motor symptoms specific to Parkinson's disease (PD) could affect the differences observed in brain age metrics, predicted age difference (PAD) between PD patients with cognitive impairment (PD-CI) and without cognitive impairment (PD-NCI), an additional ANCOVA analysis was done. Severity of motor symptoms (UPDRS part III score) was added as a covariate in addition to age, sex, and education. This subgroup comparison showed that PD-CI patients still had significantly higher levels of PAD in gray matter (GM) compared to PD-NCI patients, even after accounting for motor symptom severity (PD-CI, 5.79 ± 6.56 years; PD-NCI, 0.59 ± 7.01 years; $F_{(1,55)} = 6.53$, adjusted $P = 0.027$; Supplementary Figure 1a). However, the difference in PAD levels in white matter (WM) between the two groups was not statistically significant after the adjustment of motor symptom severity (PD-CI, 9.65 ± 7.53 years; PD-NCI, 5.98 ± 7.74 years; $F_{(1,55)} = 2.58$, adjusted $P = 0.114$; Supplementary Figure 1b). Notably, the mean PAD measures displayed were adjusted by ANCOVA given the mean values of continuous covariates (age, education, and UPDRS part III) and female class of sex covariate.

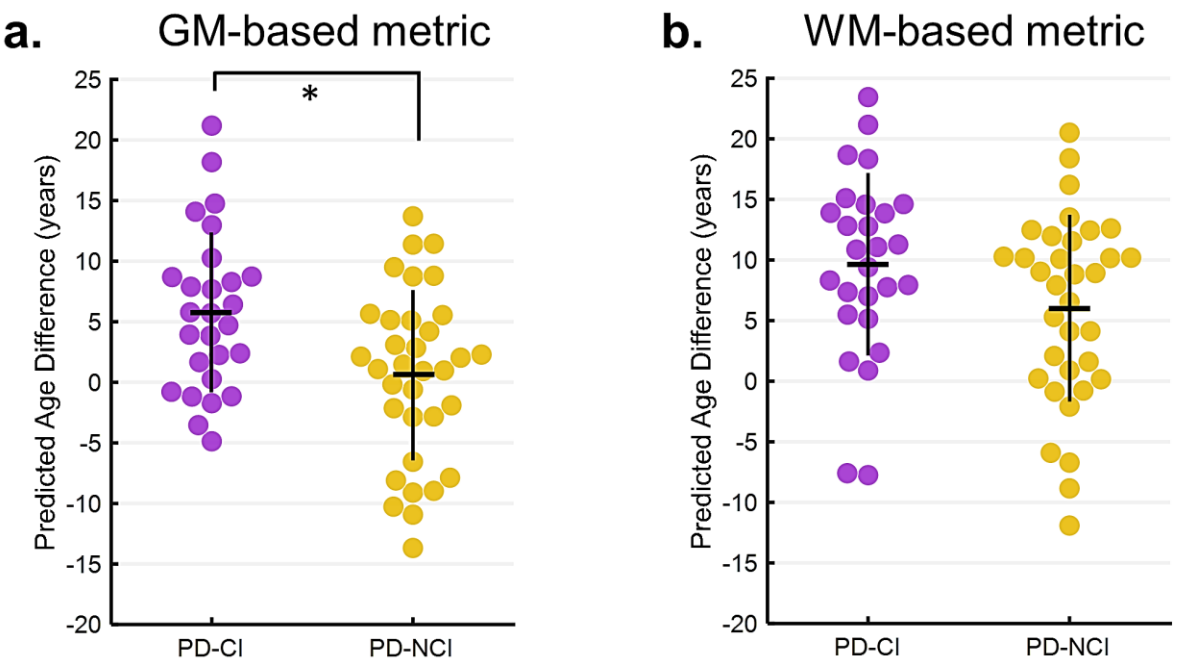

Supplementary Figure 1. Comparison of PAD measures between PD-CI and PD-NCI. Vertical and horizontal lines that overlap observation dots indicate interquartile range and median, respectively. Asterisk sign (*) indicates adjusted $P < 0.05$.

Supplementary Note 2: Image Quality Assurance

Before we performed data analysis, all T1-weighted images underwent quality assurance (QA) procedures which are included in the Computational Anatomy Toolbox 12 (CAT12; http://dbm.neuro.uni-jena.de/cat.html), a retrospective QA framework for empirical quantification of quality differences. Retrospective QA involved automatic evaluation of essential image qualities such as noise, inhomogeneity, and image resolution. These quality measures were scaled to a rating scale, and “good” image quality level was required. Additional visual inspection was conducted to examine whether artifacts, including severe motion and abnormal lesions, remained in the images. All diffusion datasets also underwent QA procedures, including examinations for the signal-to-noise ratio (SNR), degree of alignment between T1- and diffusion-weighted images, and the motion-induced signal dropout (Chen et al., 2019). The SNR was evaluated by calculating the mean signal of an object divided by the standard deviation (SD) of the background noise (Dietrich et al., 2007). In practice, the signal was determined using a central square of an image for each slice, and the noise was averaged from 4 corner regions. Diffusion datasets with an SNR higher than mean SNR minus 2.5 SDs at their site were included. The degree of within-subject alignment between T1- and diffusion-weighted images was evaluated by calculating the spatial correlation between the T1-weighted image–derived WM tissue probability map and the diffusion-weighted image–derived generalized fractional anisotropy (GFA) map. Higher spatial correlation indicated greater spatial alignment between T1- and diffusion-weighted images. In addition, because of the relatively long scan time of DSI, in-scanner head motion would

inevitably cause signal dropout in diffusion-weighted images, particularly in those with high *b* values (the magnitude of diffusion encoding gradients). For this reason, all participants lay on the MRI table with the head packed with expandable foam cushions to restrict head movement. All acquired DSI datasets (5,712 images per participant) were examined by comparing the signal in the central square of each image with the predicted signal attenuation. Signal deviation from the predicted distribution was considered signal loss. Data with more than 60 images of signal dropout per participant (1% of the total diffusion-weighted images) were discarded. Notably, a prospective visual inspection was conducted to exclude datasets presenting severe head motion or unexpected brain lesions during scanning.

Supplementary Note 3: Image Data Processing

In the image feature processing for GM, voxel-based morphometry and surface-based morphometry of 3D MPRAGE data were used. The image analyses were performed using an extension of the Statistical Parametric Mapping package (SPM12; https://www.fil.ion.ucl.ac.uk/spm/) (Ashburner et al., 2014) called Computational Anatomy Toolbox (CAT12; http://www.neuro.uni-jena.de/cat) (Gaser and Dahnke, 2016). For voxel-based morphometry analysis, we estimated regional gray matter volume by conducting the following procedures: (1) the T1-weighted images were spatially normalized and segmented into three major tissue classes (i.e. gray matter, white matter, and cerebrospinal fluid), (2) for spatial registration, the segmented tissue probability maps were taken and spatially registered onto the predefined standard templates by using the Geodesic Shooting algorithm (Ashburner and Friston, 2011), (3) the normalized maps were further modulated with the maps of Jacobian determinant to preserve the information of gray matter volumes of native space, and (4) the predefined LPBA40 probabilistic atlas containing 56 regions of interest (ROIs) (Shattuck et al., 2008) was transformed into the native space to estimate regional gray matter tissue volumes.

In addition, for surface-based morphometry analysis of cortical thickness, we applied the automated surface-preprocessing algorithms included in the CAT12 toolbox that enable the simultaneous estimation of cortical thickness of the left and right hemispheres by using the projection-based thickness method (Dahnke et al., 2013). Herein, cortical thickness was determined by estimating the WM distance based on tissue segmentation in the native space. The WM distance and a derived neighbor relationship were used to project local maxima (which is equal to the cortical thickness) onto

other GM voxels. This approach included partial volume correction and correction for sulcal blurring and sulcal asymmetries. After the initial surface reconstruction, topological defects were repaired using spherical harmonics (Yotter et al., 2011a), and this topological correction was followed by a surface refinement, which resulted in the final central surface mesh. Subsequently, the individual cortical surface mesh was then reparameterized and spatially registered to the surfaced-based template using a spherical mapping with minimal distortions (Yotter et al., 2011b). After that, the Desikan–Killiany cortical atlas (containing 68 cortical ROIs) on the template was transformed to sample mean cortical features in the native space (Desikan et al., 2006). In this manner, 56 volumetric features and 68 cortical thickness features were obtained to estimate GM-based brain age and calculate the corresponding predicted age difference (PAD).

In the image processing for WM, our in-house algorithm called tract-based automatic analysis (Chen et al., 2015) was employed. First, the diffusion indices including GFA and mean diffusivity (MD) derived from the DSI dataset were computed using the regularization version of the framework of mean apparent propagator MRI (Hsu and Tseng, 2018; Ozarslan et al., 2013). The signal in 3D diffusion-encoding space was fitted with a series expansion of Hermite basis functions, which describe diffusion in various microstructural geometries (Avram et al., 2016). The zero-order term in the expansion series contained the diffusion tensor that characterizes the Gaussian displacement distribution. Higher-order terms in the expansion series were the orthogonal corrections to the Gaussian approximation, and these were used for reconstructing the average propagator. The MD in

each voxel was determined by calculating the mean of the 3 eigenvalues of the diffusion tensor. We quantified GFA as the SD of the orientation distribution function divided by the root mean square of the orientation distribution function. To extract effective features of WM, the diffusion indices were sampled according to the spatial coordinates of 45 predefined major fiber tract bundles over the whole brain, which were constructed in the DSI template called NTU-DSI-122 (Hsu et al., 2015) through deterministic streamline-based tractography with multiple ROIs defined in the automated anatomical labeling atlas (Tzourio-Mazoyer et al., 2002). In practice, the sampling coordinates of major tract bundles were transformed from NTU-DSI-122 into individual DSI datasets with the corresponding deformation maps. The deformation maps were obtained through 2-step registration, which included anatomical information provided by the T1-weighted images and microstructural information provided by the DSI datasets (Hsu et al., 2012). The sampling coordinates were aligned with the proceeding direction of each fiber tract bundle, and diffusion indices were sampled in the native space along the sampling coordinates normalized and divided into 100 steps. Furthermore, we averaged the indices along 100 steps. Finally, 45 GFA features and 45 MD features were obtained for estimating WM-based brain age and calculating the corresponding PAD.

Supplementary Note 4: Abbreviations for Anatomical Regions in Gray Matter and White Matter

Parcellation for Gray Matter Volume		Parcellation for Gray Matter Cortical Thickness		Parcellation for White Matter Tract Bundles	
Code	ROI Name	Code	ROI Name	Code	ROI Name
L_Sup_Fron_Gy	L superior frontal gyrus	L_Banks_STS	L banks of the superior temporal sulcus	AF_L	L arcuate fasciculus
R_Sup_Fron_Gy	R superior frontal gyrus	R_Banks_STS	R banks of the superior temporal sulcus	AF_R	R arcuate fasciculus
L_Mid_Fron_Gy	L middle frontal gyrus	L_Cau_Ant_Cing	L caudal anterior cingulate	SLF_I_L	L superior longitudinal fasciculus I
R_Mid_Fron_Gy	R middle frontal gyrus	R_Cau_Ant_Cing	R caudal anterior cingulate	SLF_I_R	R superior longitudinal fasciculus I
L_Inf_Fron_Gy	L inferior frontal gyrus	L_Cau_Mid_Fron	L caudal middle frontal	SLF_II_L	L superior longitudinal fasciculus II
R_Inf_Fron_Gy	R inferior frontal gyrus	R_Cau_Mid_Fron	R caudal middle frontal	SLF_II_R	R superior longitudinal fasciculus II
L_PrC_Gy	L precentral gyrus	L_Cuneus	L cuneus	SLF_III_L	L superior longitudinal fasciculus III
R_PrC_Gy	R precentral gyrus	R_Cuneus	R cuneus	SLF_III_R	R superior longitudinal fasciculus III
L_Mid_OrbFron_Gy	L middle orbitofrontal gyrus	L_Entorhinal	L entorhinal	FAT_L	L frontal aslant tract
R_Mid_OrbFron_Gy	R middle orbitofrontal gyrus	R_Entorhinal	R entorhinal	FAT_R	R frontal aslant tract
L_Lat_OrbFron_Gy	L lateral orbitofrontal gyrus	L_Fusiform	L Fusiform	PF_L	L perpendicular fasciculus
R_Lat_OrbFron_Gy	R lateral orbitofrontal gyrus	R_Fusiform	R Fusiform	PF_R	R perpendicular fasciculus
L_Gy_Rectus	L gyrus rectus	L_Inf_Parie	L inferior parietal	CGB_L	L cingulum of main body component
R_Gy_Rectus	R gyrus rectus	R_Inf_Parie	R inferior parietal	CGB_R	R cingulum of main body component
L_PoC_Gy	L postcentral gyrus	L_Inf_Temp_Gy	L inferior temporal gyrus	CGH_L	L cingulum of hippocampal component
R_PoC_Gy	R postcentral gyrus	R_Inf_Temp_Gy	R inferior temporal gyrus	CGH_R	R cingulum of hippocampal component
L_Sup_Parie_Gy	L superior parietal gyrus	L_Isthmus_Cing	L isthmus cingulate	FX_L	L fornix
R_Sup_Parie_Gy	R superior parietal gyrus	R_Isthmus_Cing	R isthmus cingulate	FX_R	R fornix
L_SupraMar_Gy	L supramarginal gyrus	L_Lat_Occi	L lateral occipital	ST_L	L stria terminalis
R_SupraMar_Gy	R supramarginal gyrus	R_Lat_Occi	R lateral occipital	ST_R	R stria terminalis
L_Angular_Gy	L angular gyrus	L_Lat_OrbFron	L lateral orbitofrontal	UF_L	L uncinate fasciculus
R_Angular_Gy	R angular gyrus	R_Lat_OrbFron	R lateral orbitofrontal	UF_R	R uncinate fasciculus
L_PreCuneus	L precuneus	L_Lingual	L lingual	IFOF_L	L inferior frontal occipital fasciculus
R_PreCuneus	R precuneus	R_Lingual	R lingual	IFOF_R	R inferior frontal occipital fasciculus
L_Sup_Occi_Gy	L superior occipital gyrus	L_Med_OrbFron	L medial orbitofrontal	ILF_L	L inferior longitudinal fasciculus
R_Sup_Occi_Gy	R superior occipital gyrus	R_Med_OrbFron	R medial orbitofrontal	ILF_R	R inferior longitudinal fasciculus
L_Mid_Occi_Gy	L middle occipital gyrus	L_Mid_Temp_Gy	L middle temporal gyrus	FS_PFC_L	L frontal-striatum of prefrontal cortex
R_Mid_Occi_Gy	R middle occipital gyrus	R_Mid_Temp_Gy	R middle temporal gyrus	FS_PFC_R	R frontal-striatum of prefrontal cortex
L_Inf_Occi_Gy	L inferior occipital gyrus	L_ParaHipp	L parahippocampal	FS_M_L	L frontal-striatum of motor cortex
R_Inf_Occi_Gy	R inferior occipital gyrus	R_ParaHipp	R parahippocampal	FS_M_R	R frontal-striatum of motor cortex
L_Cuneus	L cuneus	L_ParaCen	L paracentral	TR_PFC_L	L thalamic radiation of prefrontal cortex
R_Cuneus	R cuneus	R_ParaCen	R paracentral	TR_PFC_R	R thalamic radiation of prefrontal cortex
L_Sup_Temp_Gy	L superior temporal gyrus	L_Pars_Oper	L pars opercularis	TR_SM_L	L thalamic radiation of sensorimotor cortex
R_Sup_Temp_Gy	R superior temporal gyrus	R_Pars_Oper	R pars opercularis	TR_SM_R	R thalamic radiation of sensorimotor cortex
L_Mid_Temp_Gy	L middle temporal gyrus	L_Pars_Orb	L pars orbitalis	TR_aud_L	L thalamic radiation of auditory part
R_Mid_Temp_Gy	R middle temporal gyrus	R_Pars_Orb	R pars orbitalis	TR_aud_R	R thalamic radiation of auditory part
L_Inf_Temp_Gy	L inferior temporal gyrus	L_Pars_Tri	L pars triangularis	TR_opt_L	L thalamic radiation of optic part
R_Inf_Temp_Gy	R inferior temporal gyrus	R_Pars_Tri	R pars triangularis	TR_opt_R	R thalamic radiation of optic part
L_ParaHipp_Gy	L parahippocampal gyrus	L_PeriCal	L pericalcarine	CST_L	L corticospinal tract
R_ParaHipp_Gy	R parahippocampal gyrus	R_PeriCal	R pericalcarine	CST_R	R corticospinal tract
L_Lingual_Gy	L lingual gyrus	L_PostCen	L postcentral	CC_genu	corpus callosum of genu
R_Lingual_Gy	R lingual gyrus	R_PostCen	R postcentral	CC_SM	corpus callosum of sensorimotor cortex
L_Fusiform_Gy	L fusiform gyrus	L_Post_Cing	L posterior cingulate	CC_pariet	corpus callosum of parietal
R_Fusiform_Gy	R fusiform gyrus	R_Post_Cing	R posterior cingulate	CC_temp	corpus callosum of temporal
L_Insular_Cortex	L insular cortex	L_PreCen	L precentral	CC_splen	corpus callosum of splenium
R_Insular_Cortex	R insular cortex	R_PreCen	R precentral		
L_Cing_Gy	L cingulate gyrus	L_PreCuneus	L precuneus		
R_Cing_Gy	R cingulate gyrus	R_PreCuneus	R precuneus		
L_Caudate	L caudate	L_Ros_Ant_Cing	L rostral anterior cingulate		
R_Caudate	R caudate	R_Ros_Ant_Cing	R rostral anterior cingulate		
L_Putamen	L putamen	L_Ros_Mid_Fron	L rostral middle frontal		
R_Putamen	R putamen	R_Ros_Mid_Fron	R rostral middle frontal		
L_Hipp	L hippocampus	L_Sup_Fron	L superior frontal		
R_Hipp	R hippocampus	R_Sup_Fron	R superior frontal		
BiLat_Cerebellum	bilateral cerebellum	L_Sup_Parie	L superior parietal		
BiLat_Brainstem	bilateral brainstem	R_Sup_Parie	R superior parietal		
		L_Sup_Temp_Gy	L superior temporal gyrus		
		R_Sup_Temp_Gy	R superior temporal gyrus		
		L_SupraMar	L supramarginal		
		R_SupraMar	R supramarginal		
		L_Fron_Pole	L frontal pole		
		R_Fron_Pole	R frontal pole		
		L_Temp_Pole	L temporal pole		
		R_Temp_Pole	R temporal pole		
		L_Trans_Temp	L transverse temporal		
		R_Trans_Temp	R transverse temporal		
		L_Insula	L insula		
		R_Insula	R insula		

Note: the anatomical parcellation was based on the following studies:

1. Gray Matter Volume: Shattuck, David W., et al. "Construction of a 3D probabilistic atlas of human cortical structures." Neuroimage 39.3 (2008): 1064-1080.
2. Gray Matter Cortical Thickness: Desikan, Rahul S., et al. "An automated labeling system for subdividing the human cerebral cortex on MRI scans into gyral based regions of interest." Neuroimage 31.3 (2006): 968-980.
3. White Matter Tract Bundles: Chen, Yu-Jen, et al. "Automatic whole brain tract-based analysis using predefined tracts in a diffusion spectrum imaging template and an accurate registration strategy." Human brain mapping 36.9 (2015): 3441-3458.

Supplementary Note 5: Performance of Brain Age Prediction Model

The brain age models used in this study were originally established in our previous study (Chen et al., 2022); we performed 10-fold cross-validation on the training set, and the brain age models showed a strong linear correlation and low MAE (in the unit of year) between chronological age and brain predicted age for the general population across the lifespan based on GM features ($\rho = 0.956$, MAE = 4.34) and WM features ($\rho = 0.944$, MAE = 4.76). The models also accurately predicted brain age in an independent test set derived from GM features ($\rho = 0.943$, MAE = 4.69) and WM features ($\rho = 0.967$, MAE = 3.95). Given that the age-related bias in the PAD measures was minimized by using additional linear correction method (de Lange and Cole, 2020), the correlation analysis of the PAD scores against chronological age showed little age-related bias in PAD measures in both of the training and test sets for the general population (GM-PAD: $\rho = -0.031$, $P = 0.495$, and $\rho = -0.096$, $P = 0.429$, respectively; WM-PAD: $\rho = -0.055$, $P = 0.226$, and $\rho = -0.006$, $P = 0.960$, respectively).

In order to detect the subtle difference between PD-CI and PD-NCI, we leveraged the notion of continual learning and performed the domain adaption procedure to the brain age models (Lenga et al., 2020), helping the models perform a better fit in the elderly population. In practice, we fine-tuned the original brain age models that were trained for general population across the lifespan to specifically fit the training data with the age range for the elderly (age > 50 years). The original brain age models can achieve acceptable performance in the elderly sample in terms of MAE and age-related bias (Supplementary Figure 2; performance metrics are displayed in each panel title). However, compared

to the performance obtained from the general population, the original brain age models seemed to slightly overfit to those in other age range. By fine-tuning the models based on the data from older age, the model performance for the brain age prediction in the elderly was significantly improved with respect to MAE and age-related bias (Supplementary Figure 3). We used the fine-tuned models to predict individual brain age for the patients and controls.

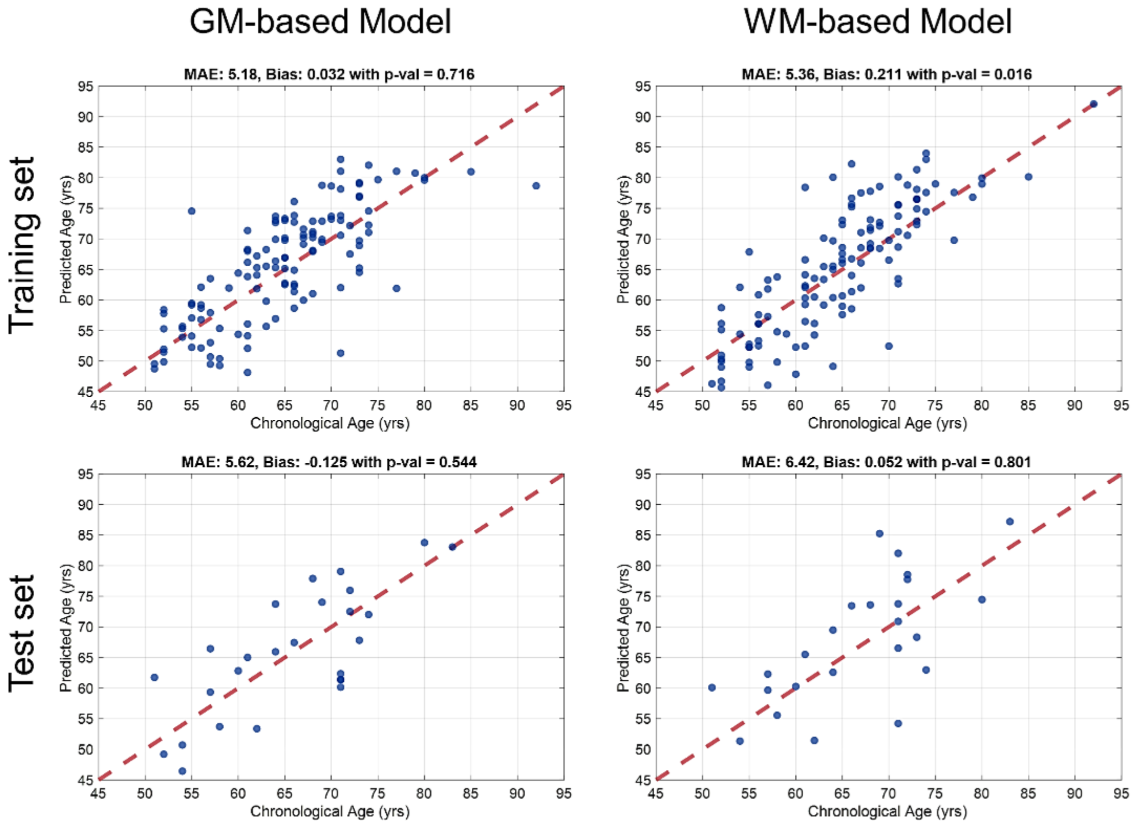

Supplementary Figure 2. The scatter plot of chronological age against predicted age made by the original universal brain age models in the training and test sets. The performance metrics, i.e. mean absolute error (MAE) and age-related bias (the correlation between predicted age difference and chronological age), are displayed in each panel title.

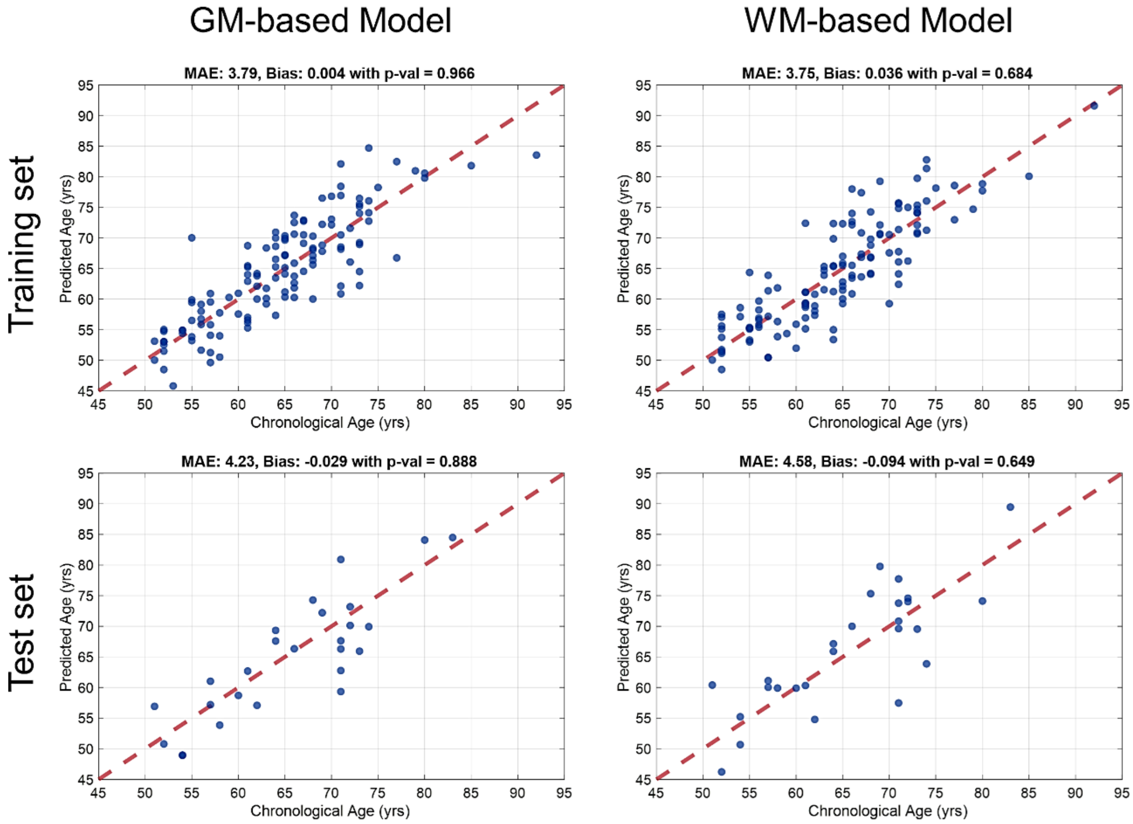

Supplementary Figure 3. The scatter plot of chronological age against predicted age made by the fine-tuned brain age models in the training and test sets. The performance metrics, i.e. mean absolute error (MAE) and age-related bias (the correlation between predicted age difference and chronological age), are displayed in each panel title.

Supplementary Table 1

Gray matter feature differences in PD-CI vs. HC (healthy controls)

Serial	Measure	ROI	PD-CI	PD-NCI	HC	t-value	p-value
1	Vol	L Sup Fron Gy	26.450	27.627	28.376	-2.427	0.017
2	Vol	R Sup Fron Gy	25.888	26.630	27.677	-2.429	0.017
5	Vol	L Inf Fron Gy	9.739	10.679	10.778	-3.092	0.003
7	Vol	L PrC Gy	11.147	11.725	12.174	-2.535	0.013
8	Vol	R PrC Gy	10.755	11.152	11.541	-2.046	0.044
9	Vol	L Mid OrbFron Gy	4.393	4.636	4.753	-2.498	0.014
10	Vol	R Mid OrbFron Gy	4.655	4.950	5.086	-2.935	0.004
11	Vol	L Lat OrbFron Gy	2.928	3.155	3.309	-3.047	0.003
12	Vol	R Lat OrbFron Gy	2.646	2.909	2.991	-3.690	0.000
15	Vol	L PoC Gy	9.111	9.662	10.126	-3.222	0.002
16	Vol	R PoC Gy	8.534	8.903	9.522	-3.206	0.002
17	Vol	L Sup Parie Gy	11.657	12.261	12.682	-2.581	0.011
18	Vol	R Sup Parie Gy	11.418	12.113	12.609	-3.038	0.003
19	Vol	L SupraMar Gy	7.450	8.005	8.292	-3.020	0.003
20	Vol	R SupraMar Gy	7.342	7.481	7.820	-2.078	0.041
21	Vol	L Angular Gy	9.703	10.385	10.556	-2.521	0.013
23	Vol	L PreCuneus	6.365	6.898	7.194	-3.549	0.001
24	Vol	R PreCuneus	6.486	6.904	7.042	-2.484	0.015
26	Vol	R Sup Occi Gy	4.057	4.350	4.513	-2.693	0.008
28	Vol	R Mid Occi Gy	11.063	11.807	12.030	-2.424	0.017
30	Vol	R Inf Occi Gy	5.698	5.954	6.096	-2.039	0.044
31	Vol	L Cuneus	2.973	3.146	3.286	-2.063	0.042
32	Vol	R Cuneus	3.290	3.622	3.627	-1.988	0.050
33	Vol	L Sup Temp Gy	14.343	14.904	15.812	-3.164	0.002
34	Vol	R Sup Temp Gy	13.487	14.108	14.546	-2.674	0.009
36	Vol	R Mid Temp Gy	13.436	14.104	14.398	-2.187	0.031
37	Vol	L Inf Temp Gy	10.709	11.243	11.804	-2.989	0.004
38	Vol	R Inf Temp Gy	11.325	11.899	12.697	-3.631	0.000
39	Vol	L ParaHipp Gy	3.256	3.599	3.677	-4.403	0.000
40	Vol	R ParaHipp Gy	3.519	3.816	3.871	-3.412	0.001
41	Vol	L Lingual Gy	6.664	7.108	7.414	-2.913	0.005
42	Vol	R Lingual Gy	6.675	7.105	7.466	-3.366	0.001

43	Vol	L Fusiform Gy	6.550	6.962	7.189	-2.752	0.007
44	Vol	R Fusiform Gy	6.562	6.879	7.082	-2.460	0.016
45	Vol	L Insular Cortex	5.233	5.411	5.632	-2.615	0.011
46	Vol	R Insular Cortex	5.203	5.355	5.518	-2.097	0.039
47	Vol	L Cing Gy	6.793	7.105	7.311	-2.110	0.038
48	Vol	R Cing Gy	7.703	8.014	8.247	-2.168	0.033
49	Vol	L Caudate	2.590	2.872	3.075	-3.635	0.000
50	Vol	R Caudate	2.375	2.732	2.926	-3.800	0.000
51	Vol	L Putamen	3.021	3.486	3.570	-2.850	0.005
52	Vol	R Putamen	3.105	3.391	3.513	-2.311	0.023
53	Vol	L Hipp	2.999	3.311	3.482	-4.473	0.000
54	Vol	R Hipp	3.129	3.444	3.560	-3.666	0.000
59	CT	L Cau Ant Cing	2.327	2.480	2.516	-2.288	0.025
61	CT	L Cau Mid Fron	2.639	2.749	2.791	-3.078	0.003
62	CT	R Cau Mid Fron	2.661	2.761	2.799	-3.055	0.003
63	CT	L Cuneus	1.796	1.858	1.960	-3.682	0.000
64	CT	R Cuneus	1.788	1.845	1.932	-3.353	0.001
65	CT	L Entorhinal	3.078	3.466	3.532	-3.149	0.002
66	CT	R Entorhinal	3.221	3.666	3.631	-2.602	0.011
67	CT	L Fusiform	2.579	2.668	2.706	-2.505	0.014
68	CT	R Fusiform	2.617	2.684	2.725	-2.150	0.034
70	CT	R Inf Parie	2.485	2.600	2.577	-2.078	0.041
71	CT	L Inf Temp Gy	2.725	2.809	2.863	-2.577	0.012
72	CT	R Inf Temp Gy	2.739	2.849	2.886	-2.654	0.009
73	CT	L Isthmus Cing	2.218	2.295	2.398	-2.657	0.009
74	CT	R Isthmus Cing	2.222	2.310	2.344	-2.117	0.037
75	CT	L Lat Occi	2.054	2.158	2.219	-3.647	0.000
76	CT	R Lat Occi	2.061	2.137	2.219	-3.425	0.001
77	CT	L Lat OrbFron	2.658	2.771	2.778	-2.153	0.034
79	CT	L Lingual	1.891	1.898	1.980	-2.025	0.046
80	CT	R Lingual	1.909	1.930	2.019	-2.847	0.005
81	CT	L Med OrbFron	2.473	2.559	2.566	-2.044	0.044
83	CT	L Mid Temp Gy	2.876	2.988	3.004	-2.392	0.019
87	CT	L ParaCen	2.269	2.348	2.431	-2.628	0.010
88	CT	R ParaCen	2.278	2.325	2.425	-2.610	0.011
89	CT	L Pars Oper	2.747	2.847	2.880	-2.637	0.010
92	CT	R Pars Orb	2.630	2.781	2.740	-2.071	0.041
95	CT	L PeriCal	1.663	1.700	1.804	-2.660	0.009

97	CT	L PostCen	1.998	2.093	2.119	-2.653	0.009
98	CT	R PostCen	2.044	2.126	2.137	-2.022	0.046
99	CT	L Post Cing	2.312	2.433	2.507	-3.874	0.000
100	CT	R Post Cing	2.353	2.442	2.476	-2.749	0.007
101	CT	L PreCen	2.319	2.410	2.449	-2.066	0.042
103	CT	L PreCuneus	2.426	2.509	2.530	-2.876	0.005
107	CT	L Ros Mid Fron	2.452	2.584	2.583	-3.012	0.003
111	CT	L Sup Parie	2.225	2.316	2.322	-2.195	0.031
113	CT	L Sup Temp Gy	2.710	2.790	2.839	-2.895	0.005
114	CT	R Sup Temp Gy	2.736	2.851	2.837	-2.100	0.039
115	CT	L SupraMar	2.583	2.696	2.676	-2.155	0.034
120	CT	R Temp Pole	3.221	3.442	3.531	-2.749	0.007
121	CT	L Trans Temp	2.136	2.198	2.311	-2.886	0.005
123	CT	L Insula	3.254	3.378	3.456	-3.201	0.002

Gray matter feature differences in PD-NCI vs. HC

Serial	Measure	ROI	PD-CI	PD-NCI	HC	t-value	p-value
16	Vol	R PoC Gy	8.534	8.903	9.522	-2.351	0.021
33	Vol	L Sup Temp Gy	14.343	14.904	15.812	-2.289	0.024
38	Vol	R Inf Temp Gy	11.325	11.899	12.697	-2.473	0.015
63	CT	L Cuneus	1.796	1.858	1.960	-2.685	0.009
64	CT	R Cuneus	1.788	1.845	1.932	-2.373	0.020
76	CT	R Lat Occi	2.061	2.137	2.219	-2.074	0.041
79	CT	L Lingual	1.891	1.898	1.980	-2.181	0.032
80	CT	R Lingual	1.909	1.930	2.019	-2.684	0.009
88	CT	R ParaCen	2.278	2.325	2.425	-2.073	0.041
95	CT	L PeriCal	1.663	1.700	1.804	-2.285	0.025
121	CT	L Trans Temp	2.136	2.198	2.311	-2.176	0.032

Gray matter feature differences in PD-CI vs. PD-NCI

Serial	Measure	ROI	PD-CI	PD-NCI	HC	t-value	p-value
5	Vol	L Inf Fron Gy	9.739	10.679	10.778	-3.253	0.002

10	Vol	R Mid OrbFron Gy	4.655	4.950	5.086	-2.335	0.022
11	Vol	L Lat OrbFron Gy	2.928	3.155	3.309	-2.113	0.037
12	Vol	R Lat OrbFron Gy	2.646	2.909	2.991	-3.269	0.002
15	Vol	L PoC Gy	9.111	9.662	10.126	-2.033	0.045
18	Vol	R Sup Parie Gy	11.418	12.113	12.609	-2.061	0.042
19	Vol	L SupraMar Gy	7.450	8.005	8.292	-2.316	0.023
21	Vol	L Angular Gy	9.703	10.385	10.556	-2.344	0.021
23	Vol	L PreCuneus	6.365	6.898	7.194	-2.658	0.009
24	Vol	R PreCuneus	6.486	6.904	7.042	-2.172	0.033
26	Vol	R Sup Occi Gy	4.057	4.350	4.513	-2.012	0.047
28	Vol	R Mid Occi Gy	11.063	11.807	12.030	-2.169	0.033
32	Vol	R Cuneus	3.290	3.622	3.627	-2.282	0.025
35	Vol	L Mid Temp Gy	13.297	14.080	14.108	-2.051	0.043
39	Vol	L ParaHipp Gy	3.256	3.599	3.677	-4.172	0.000
40	Vol	R ParaHipp Gy	3.519	3.816	3.871	-3.343	0.001
41	Vol	L Lingual Gy	6.664	7.108	7.414	-2.008	0.048
42	Vol	R Lingual Gy	6.675	7.105	7.466	-2.128	0.036
43	Vol	L Fusiform Gy	6.550	6.962	7.189	-2.064	0.042
49	Vol	L Caudate	2.590	2.872	3.075	-2.459	0.016
50	Vol	R Caudate	2.375	2.732	2.926	-2.870	0.005
51	Vol	L Putamen	3.021	3.486	3.570	-2.807	0.006
53	Vol	L Hipp	2.999	3.311	3.482	-3.359	0.001
54	Vol	R Hipp	3.129	3.444	3.560	-3.117	0.002
59	CT	L Cau Ant Cing	2.327	2.480	2.516	-2.150	0.034
60	CT	R Cau Ant Cing	2.373	2.537	2.475	-2.826	0.006
61	CT	L Cau Mid Fron	2.639	2.749	2.791	-2.582	0.011
62	CT	R Cau Mid Fron	2.661	2.761	2.799	-2.570	0.012
65	CT	L Entorhinal	3.078	3.466	3.532	-3.130	0.002
66	CT	R Entorhinal	3.221	3.666	3.631	-3.285	0.001
67	CT	L Fusiform	2.579	2.668	2.706	-2.037	0.045
69	CT	L Inf Parie	2.489	2.592	2.571	-2.602	0.011
70	CT	R Inf Parie	2.485	2.600	2.577	-3.009	0.003
72	CT	R Inf Temp Gy	2.739	2.849	2.886	-2.294	0.024
75	CT	L Lat Occi	2.054	2.158	2.219	-2.683	0.009
77	CT	L Lat OrbFron	2.658	2.771	2.778	-2.354	0.021
81	CT	L Med OrbFron	2.473	2.559	2.566	-2.190	0.031
82	CT	R Med OrbFron	2.530	2.623	2.620	-2.305	0.024
83	CT	L Mid Temp Gy	2.876	2.988	3.004	-2.441	0.017

89	CT	L Pars Oper	2.747	2.847	2.880	-2.305	0.024
92	CT	R Pars Orb	2.630	2.781	2.740	-3.291	0.001
93	CT	L Pars Tri	2.617	2.723	2.691	-2.726	0.008
94	CT	R Pars Tri	2.615	2.735	2.696	-3.077	0.003
97	CT	L PostCen	1.998	2.093	2.119	-2.404	0.018
98	CT	R PostCen	2.044	2.126	2.137	-2.076	0.041
99	CT	L Post Cing	2.312	2.433	2.507	-2.793	0.006
100	CT	R Post Cing	2.353	2.442	2.476	-2.326	0.022
103	CT	L PreCuneus	2.426	2.509	2.530	-2.660	0.009
104	CT	R PreCuneus	2.478	2.546	2.549	-2.004	0.048
107	CT	L Ros Mid Fron	2.452	2.584	2.583	-3.528	0.001
108	CT	R Ros Mid Fron	2.479	2.583	2.564	-2.803	0.006
109	CT	L Sup Fron	2.818	2.906	2.904	-2.081	0.040
111	CT	L Sup Parie	2.225	2.316	2.322	-2.407	0.018
112	CT	R Sup Parie	2.238	2.313	2.306	-2.201	0.030
113	CT	L Sup Temp Gy	2.710	2.790	2.839	-2.092	0.039
114	CT	R Sup Temp Gy	2.736	2.851	2.837	-2.775	0.007
115	CT	L SupraMar	2.583	2.696	2.676	-3.027	0.003
116	CT	R SupraMar	2.608	2.693	2.631	-2.554	0.012
120	CT	R Temp Pole	3.221	3.442	3.531	-2.274	0.025
123	CT	L Insula	3.254	3.378	3.456	-2.293	0.024

Supplementary Table 2

White matter feature differences in PD-CI vs. HC

Serial	Measure	ROI	PD-CI	PD-NCI	HC	t-value	p-value
1	GFA	AF L	0.449	0.472	0.472	-1.996	0.049
2	GFA	AF R	0.436	0.452	0.460	-2.226	0.029
3	GFA	SLF I L	0.487	0.518	0.513	-2.241	0.028
5	GFA	SLF II L	0.437	0.459	0.473	-2.744	0.007
6	GFA	SLF II R	0.456	0.474	0.485	-2.604	0.011
7	GFA	SLF III L	0.458	0.479	0.494	-2.488	0.015
8	GFA	SLF III R	0.426	0.439	0.456	-2.334	0.022
9	GFA	FAT L	0.461	0.493	0.499	-2.759	0.007
10	GFA	FAT R	0.403	0.427	0.435	-2.443	0.017
12	GFA	PF R	0.323	0.340	0.350	-3.078	0.003
13	GFA	CGB L	0.489	0.513	0.535	-2.907	0.005
15	GFA	CGH L	0.404	0.435	0.431	-2.518	0.014
16	GFA	CGH R	0.456	0.480	0.480	-2.543	0.013
17	GFA	FX L	0.307	0.314	0.328	-2.265	0.026
18	GFA	FX R	0.351	0.368	0.368	-2.019	0.047
19	GFA	ST L	0.326	0.342	0.345	-2.678	0.009
21	GFA	UF L	0.333	0.343	0.361	-3.150	0.002
22	GFA	UF R	0.343	0.342	0.363	-2.900	0.005
23	GFA	IFOF L	0.437	0.463	0.476	-3.283	0.001
24	GFA	IFOF R	0.425	0.443	0.448	-2.226	0.029
25	GFA	ILF L	0.392	0.404	0.418	-2.002	0.048
27	GFA	FS PFC L	0.395	0.418	0.429	-3.070	0.003
28	GFA	FS PFC R	0.396	0.417	0.423	-2.278	0.025
29	GFA	FS M L	0.464	0.488	0.494	-2.544	0.013
31	GFA	TR PFC L	0.455	0.482	0.491	-3.356	0.001
32	GFA	TR PFC R	0.455	0.481	0.480	-2.106	0.038
33	GFA	TR SM L	0.480	0.506	0.515	-2.812	0.006
35	GFA	TR aud L	0.348	0.363	0.365	-2.091	0.039
37	GFA	TR opt L	0.437	0.463	0.475	-3.087	0.003
38	GFA	TR opt R	0.451	0.473	0.476	-2.107	0.038
39	GFA	CST L	0.599	0.624	0.631	-3.092	0.003
40	GFA	CST R	0.603	0.625	0.627	-2.266	0.026

41	GFA	CC genu	0.438	0.474	0.484	-3.537	0.001
42	GFA	CC SM	0.506	0.553	0.556	-3.200	0.002
43	GFA	CC pariet	0.463	0.503	0.507	-3.356	0.001
44	GFA	CC temp	0.411	0.436	0.440	-2.823	0.006
45	GFA	CC splen	0.456	0.507	0.507	-3.770	0.000
46	MD	AF L	0.591	0.546	0.549	3.098	0.003
47	MD	AF R	0.580	0.536	0.527	3.452	0.001
48	MD	SLF I L	0.597	0.554	0.558	2.354	0.021
50	MD	SLF II L	0.577	0.517	0.512	3.489	0.001
51	MD	SLF II R	0.563	0.527	0.507	3.397	0.001
52	MD	SLF III L	0.548	0.499	0.493	3.145	0.002
53	MD	SLF III R	0.569	0.545	0.533	2.274	0.025
54	MD	FAT L	0.591	0.537	0.539	2.998	0.004
55	MD	FAT R	0.710	0.646	0.620	3.664	0.000
56	MD	PF L	0.774	0.729	0.725	2.200	0.030
57	MD	PF R	0.762	0.727	0.687	3.331	0.001
61	MD	CGH R	0.711	0.638	0.661	2.241	0.028
62	MD	FX L	2.968	2.577	2.443	4.848	0.000
63	MD	FX R	2.057	1.860	1.739	4.241	0.000
64	MD	ST L	2.888	2.694	2.552	3.835	0.000
65	MD	ST R	2.364	2.237	2.127	3.505	0.001
66	MD	UF L	0.750	0.698	0.663	4.397	0.000
67	MD	UF R	0.765	0.713	0.686	5.166	0.000
68	MD	IFOF L	0.709	0.652	0.645	2.376	0.020
69	MD	IFOF R	0.687	0.638	0.635	2.579	0.012
70	MD	ILF L	0.703	0.648	0.630	2.157	0.034
71	MD	ILF R	0.681	0.623	0.612	2.585	0.011
72	MD	FS PFC L	0.651	0.587	0.587	2.661	0.009
74	MD	FS M L	0.596	0.547	0.544	2.834	0.006
75	MD	FS M R	0.574	0.543	0.532	2.254	0.027
76	MD	TR PFC L	0.635	0.579	0.581	2.708	0.008
77	MD	TR PFC R	0.635	0.579	0.589	2.047	0.044
79	MD	TR SM R	0.607	0.550	0.557	2.270	0.026
80	MD	TR aud L	0.774	0.709	0.698	3.346	0.001
82	MD	TR opt L	0.947	0.842	0.815	2.450	0.016
84	MD	CST L	0.584	0.529	0.520	3.810	0.000
85	MD	CST R	0.612	0.566	0.566	2.428	0.017
86	MD	CC genu	0.780	0.688	0.695	2.572	0.012

88	MD	CC pariet	1.086	0.958	0.964	2.091	0.039
89	MD	CC temp	1.794	1.583	1.497	4.406	0.000

White matter feature differences in PD-NCI vs. HC

Serial	Measure	ROI	PD-CI	PD-NCI	HC	t-value	p-value
21	GFA	UF L	0.333	0.343	0.361	-2.332	0.022
22	GFA	UF R	0.343	0.342	0.363	-3.655	0.000
57	MD	PF R	0.762	0.727	0.687	2.085	0.040
66	MD	UF L	0.750	0.698	0.663	2.103	0.038
67	MD	UF R	0.765	0.713	0.686	2.048	0.044

White matter feature differences in PD-CI vs. PD-NCI

Serial	Measure	ROI	PD-CI	PD-NCI	HC	t-value	p-value
1	GFA	AF L	0.449	0.472	0.472	-2.276	0.025
3	GFA	SLF I L	0.487	0.518	0.513	-3.059	0.003
9	GFA	FAT L	0.461	0.493	0.499	-2.759	0.007
10	GFA	FAT R	0.403	0.427	0.435	-2.123	0.037
12	GFA	PF R	0.323	0.340	0.350	-2.246	0.027
14	GFA	CGB R	0.519	0.550	0.548	-2.292	0.024
15	GFA	CGH L	0.404	0.435	0.431	-3.363	0.001
16	GFA	CGH R	0.456	0.480	0.480	-2.981	0.004
18	GFA	FX R	0.351	0.368	0.368	-2.456	0.016
19	GFA	ST L	0.326	0.342	0.345	-2.589	0.011
23	GFA	IFOF L	0.437	0.463	0.476	-2.538	0.013
24	GFA	IFOF R	0.425	0.443	0.448	-1.995	0.049
27	GFA	FS PFC L	0.395	0.418	0.429	-2.445	0.016
28	GFA	FS PFC R	0.396	0.417	0.423	-2.050	0.043
29	GFA	FS M L	0.464	0.488	0.494	-2.303	0.024
31	GFA	TR PFC L	0.455	0.482	0.491	-2.992	0.004
32	GFA	TR PFC R	0.455	0.481	0.480	-2.610	0.011
33	GFA	TR SM L	0.480	0.506	0.515	-2.373	0.020
34	GFA	TR SM R	0.507	0.533	0.533	-2.171	0.033

35	GFA	TR aud L	0.348	0.363	0.365	-2.047	0.044
37	GFA	TR opt L	0.437	0.463	0.475	-2.503	0.014
38	GFA	TR opt R	0.451	0.473	0.476	-2.198	0.031
39	GFA	CST L	0.599	0.624	0.631	-2.825	0.006
40	GFA	CST R	0.603	0.625	0.627	-2.398	0.019
41	GFA	CC genu	0.438	0.474	0.484	-3.176	0.002
42	GFA	CC SM	0.506	0.553	0.556	-3.538	0.001
43	GFA	CC pariet	0.463	0.503	0.507	-3.484	0.001
44	GFA	CC temp	0.411	0.436	0.440	-2.839	0.006
45	GFA	CC splen	0.456	0.507	0.507	-4.408	0.000
46	MD	AF L	0.591	0.546	0.549	3.840	0.000
47	MD	AF R	0.580	0.536	0.527	3.302	0.001
48	MD	SLF I L	0.597	0.554	0.558	3.022	0.003
49	MD	SLF I R	0.595	0.549	0.564	2.125	0.036
50	MD	SLF II L	0.577	0.517	0.512	3.738	0.000
51	MD	SLF II R	0.563	0.527	0.507	2.579	0.012
52	MD	SLF III L	0.548	0.499	0.493	3.236	0.002
54	MD	FAT L	0.591	0.537	0.539	3.626	0.000
55	MD	FAT R	0.710	0.646	0.620	3.033	0.003
56	MD	PF L	0.774	0.729	0.725	2.336	0.022
60	MD	CGH L	0.696	0.635	0.655	3.173	0.002
61	MD	CGH R	0.711	0.638	0.661	3.806	0.000
62	MD	FX L	2.968	2.577	2.443	4.194	0.000
63	MD	FX R	2.057	1.860	1.739	3.058	0.003
64	MD	ST L	2.888	2.694	2.552	2.581	0.012
65	MD	ST R	2.364	2.237	2.127	2.173	0.032
66	MD	UF L	0.750	0.698	0.663	3.025	0.003
67	MD	UF R	0.765	0.713	0.686	3.974	0.000
68	MD	IFOF L	0.709	0.652	0.645	2.469	0.015
69	MD	IFOF R	0.687	0.638	0.635	2.815	0.006
71	MD	ILF R	0.681	0.623	0.612	2.525	0.013
72	MD	FS PFC L	0.651	0.587	0.587	3.096	0.003
73	MD	FS PFC R	0.647	0.583	0.595	2.829	0.006
74	MD	FS M L	0.596	0.547	0.544	3.077	0.003
76	MD	TR PFC L	0.635	0.579	0.581	3.306	0.001
77	MD	TR PFC R	0.635	0.579	0.589	2.883	0.005
78	MD	TR SM L	0.666	0.576	0.596	2.913	0.005
79	MD	TR SM R	0.607	0.550	0.557	2.968	0.004

80	MD	TR aud L	0.774	0.709	0.698	3.338	0.001
81	MD	TR aud R	0.741	0.687	0.689	2.025	0.046
82	MD	TR opt L	0.947	0.842	0.815	2.279	0.025
83	MD	TR opt R	0.817	0.757	0.759	2.304	0.024
84	MD	CST L	0.584	0.529	0.520	3.817	0.000
85	MD	CST R	0.612	0.566	0.566	2.791	0.006
86	MD	CC genu	0.780	0.688	0.695	3.227	0.002
87	MD	CC SM	0.832	0.722	0.752	2.231	0.028
88	MD	CC pariet	1.086	0.958	0.964	2.565	0.012
89	MD	CC temp	1.794	1.583	1.497	3.645	0.000
90	MD	CC splen	0.746	0.654	0.680	3.023	0.003

Supplementary References

Ashburner, J., Barnes, G., Chen, C.-C., Daunizeau, J., Flandin, G., Friston, K., Kiebel, S., Kilner, J., Litvak, V., Moran, R., 2014. SPM12 manual. Wellcome Trust Centre for Neuroimaging, London, UK 2464, 4.

Ashburner, J., Friston, K.J., 2011. Diffeomorphic registration using geodesic shooting and Gauss-Newton optimisation. NeuroImage 55, 954-967.

Avram, A.V., Sarlls, J.E., Barnett, A.S., Özarslan, E., Thomas, C., Irfanoglu, M.O., Hutchinson, E., Pierpaoli, C., Basser, P.J., 2016. Clinical feasibility of using mean apparent propagator (MAP) MRI to characterize brain tissue microstructure. NeuroImage 127, 422-434.

Chen, C.-L., Kuo, M.-C., Wu, W.-C., Hsu, Y.-C., Wu, R.-M., Tseng, W.-Y.I., 2022. Advanced brain aging in multiple system atrophy compared to Parkinson's disease. NeuroImage: Clinical 34, 102997.

Chen, C.L., Shih, Y.C., Liou, H.H., Hsu, Y.C., Lin, F.H., Tseng, W.I., 2019. Premature white matter aging in patients with right mesial temporal lobe epilepsy: A machine learning approach based on diffusion MRI data. Neuroimage Clin 24, 102033.

Chen, Y.J., Lo, Y.C., Hsu, Y.C., Fan, C.C., Hwang, T.J., Liu, C.M., Chien, Y.L., Hsieh, M.H., Liu, C.C., Hwu, H.G., Tseng, W.Y., 2015. Automatic whole brain tract-based analysis using predefined tracts in a diffusion spectrum imaging template and an accurate registration strategy. Hum Brain Mapp 36, 3441-3458.

Dahnke, R., Yotter, R.A., Gaser, C., 2013. Cortical thickness and central surface estimation. NeuroImage 65, 336-348.

de Lange, A.G., Cole, J.H., 2020. Commentary: Correction procedures in brain-age prediction. Neuroimage Clin 26, 102229.

Desikan, R.S., Ségonne, F., Fischl, B., Quinn, B.T., Dickerson, B.C., Blacker, D., Buckner, R.L., Dale, A.M., Maguire, R.P., Hyman, B.T., 2006. An automated labeling system for subdividing the human cerebral cortex on MRI scans into gyral based regions of interest. NeuroImage 31, 968-980.

Dietrich, O., Raya, J.G., Reeder, S.B., Reiser, M.F., Schoenberg, S.O., 2007. Measurement of signal-to-noise ratios in MR images: influence of multichannel coils, parallel imaging, and reconstruction filters. Journal of Magnetic Resonance Imaging: An Official Journal of the International Society for Magnetic Resonance in Medicine 26, 375-385.

Gaser, C., Dahnke, R., 2016. CAT-a computational anatomy toolbox for the analysis of structural MRI data. HBM 2016, 336-348.

Hsu, Y.C., Hsu, C.H., Tseng, W.Y., 2012. A large deformation diffeomorphic metric mapping solution for diffusion spectrum imaging datasets. Neuroimage 63, 818-834.

Hsu, Y.C., Lo, Y.C., Chen, Y.J., Wedeen, V.J., Isaac Tseng, W.Y., 2015. NTU-DSI-122: A diffusion spectrum imaging template with high anatomical matching to the ICBM-152 space. Hum Brain

Mapp 36, 3528-3541.
Hsu, Y.C., Tseng, W.Y., 2018. An efficient regularization method for diffusion MAP-MRI estimation. 2018 ISMRM-ESMRMB Joint Annual Meeting.
Lenga, M., Schulz, H., Saalbach, A., 2020. Continual learning for domain adaptation in chest x-ray classification. Medical Imaging with Deep Learning. PMLR, pp. 413-423.
Ozarslan, E., Koay, C.G., Shepherd, T.M., Komlosh, M.E., Irfanoglu, M.O., Pierpaoli, C., Basser, P.J., 2013. Mean apparent propagator (MAP) MRI: a novel diffusion imaging method for mapping tissue microstructure. NeuroImage 78, 16-32.
Shattuck, D.W., Mirza, M., Adisetiyo, V., Hojatkashani, C., Salamon, G., Narr, K.L., Poldrack, R.A., Bilder, R.M., Toga, A.W., 2008. Construction of a 3D probabilistic atlas of human cortical structures. NeuroImage 39, 1064-1080.
Tzourio-Mazoyer, N., Landeau, B., Papathanassiou, D., Crivello, F., Etard, O., Delcroix, N., Mazoyer, B., Joliot, M., 2002. Automated anatomical labeling of activations in SPM using a macroscopic anatomical parcellation of the MNI MRI single-subject brain. Neuroimage 15, 273-289.
Yotter, R.A., Dahnke, R., Thompson, P.M., Gaser, C., 2011a. Topological correction of brain surface meshes using spherical harmonics. Human brain mapping 32, 1109-1124.
Yotter, R.A., Nenadic, I., Ziegler, G., Thompson, P.M., Gaser, C., 2011b. Local cortical surface complexity maps from spherical harmonic reconstructions. Neuroimage 56, 961-973.
