# Supplementary material for: Advanced brain aging in Parkinson’s disease with cognitive impairment
Source: NPJ Parkinsons Dis. 2024 Mar 16;10:62. doi: 10.1038/s41531-024-00673-7 (PMC10944471; doi:10.1038/s41531-024-00673-7)
Supplement: Supplementary file 2 — Reporting summary [file 41531_2024_673_MOESM2_ESM.pdf]

nature portfolio

Corresponding author(s): Ming-Che Kuo & Wen-Yih Isaac Tseng

Last updated by author(s): 2024/02/23

Reporting Summary

Nature Portfolio wishes to improve the reproducibility of the work that we publish. This form provides structure for consistency and transparency in reporting. For further information on Nature Portfolio policies, see our Editorial Policies and the Editorial Policy Checklist.

Statistics

For all statistical analyses, confirm that the following items are present in the figure legend, table legend, main text, or Methods section.

n/a	Confirmed	
☐	☒	The exact sample size (n) for each experimental group/condition, given as a discrete number and unit of measurement
☐	☒	A statement on whether measurements were taken from distinct samples or whether the same sample was measured repeatedly
☐	☒	The statistical test(s) used AND whether they are one- or two-sided<br>*Only common tests should be described solely by name; describe more complex techniques in the Methods section.*
☐	☒	A description of all covariates tested
☐	☒	A description of any assumptions or corrections, such as tests of normality and adjustment for multiple comparisons
☐	☒	A full description of the statistical parameters including central tendency (e.g. means) or other basic estimates (e.g. regression coefficient) AND variation (e.g. standard deviation) or associated estimates of uncertainty (e.g. confidence intervals)
☐	☒	For null hypothesis testing, the test statistic (e.g. F, t, r) with confidence intervals, effect sizes, degrees of freedom and P value noted<br>*Give P values as exact values whenever suitable.*
☒	☐	For Bayesian analysis, information on the choice of priors and Markov chain Monte Carlo settings
☐	☒	For hierarchical and complex designs, identification of the appropriate level for tests and full reporting of outcomes
☒	☐	Estimates of effect sizes (e.g. Cohen's d, Pearson's r), indicating how they were calculated

Our web collection on statistics for biologists contains articles on many of the points above.

Software and code

Policy information about availability of computer code

Data collection	All brain images used in this study were acquired using a 3-Tesla MRI scanner (Tim Trio; Siemens, Erlangen, Germany) with a 32-channel phased-array head coil at the National Taiwan University Hospital.
Data analysis	For structural MRI data analysis, the open-access software CAT12 was used (Gaser, C., Dahnke, R., Thompson, P. M., Kurth, F. & Luders, E. CAT-a computational anatomy toolbox for the analysis of structural MRI data. BioRxiv, 2022.2006. 2011.495736 (2022)). For diffusion MRI data analysis, we used our well-developed algorithm that has been published and well-evaluated in our previous literature Chen, Y. J. et al. Automatic whole brain tract-based analysis using predefined tracts in a diffusion spectrum imaging template and an accurate registration strategy. Human brain mapping 36, 3441-3458 (2015). For statistical analysis, we used statistical and machine learning toolbox built in MATLAB R2022a.

For manuscripts utilizing custom algorithms or software that are central to the research but not yet described in published literature, software must be made available to editors and reviewers. We strongly encourage code deposition in a community repository (e.g. GitHub). See the Nature Portfolio guidelines for submitting code & software for further information.

Data

Policy information about availability of data

All manuscripts must include a data availability statement. This statement should provide the following information, where applicable:

- Accession codes, unique identifiers, or web links for publicly available datasets
- A description of any restrictions on data availability
- For clinical datasets or third party data, please ensure that the statement adheres to our policy

The raw neuroimaging data acquired from National Taiwan University Hospital (NTUH) are not available due to confidentiality agreement of NTUH Research Ethics Committee. The secondary data are conditionally available upon request from the corresponding author. The methodologies of brain age modeling and analytic scrips are available in our published paper including the URL to the online open-access repository (Chen, C. L. et al. Generalization of diffusion magnetic resonance imaging-based brain age prediction model through transfer learning. Neuroimage 217, 116831, doi:10.1016/j.neuroimage.2020.116831 (2020)). Code of imaging process is conditionally available upon request from the corresponding author.

Research involving human participants, their data, or biological material

Policy information about studies with human participants or human data. See also policy information about sex, gender (identity/presentation), and sexual orientation and race, ethnicity and racism.

Reporting on sex and gender	The term sex (biological attribute) was used throughout the manuscript. This information was acquired from the participants' medical history as well as their self-report. The details of sex information has been described in the manuscript.
Reporting on race, ethnicity, or other socially relevant groupings	All participants acquired in this study at National Taiwan University Hospital (NTUH) were Asian.
Population characteristics	This study enrolled the patients with PD-CI and PD-NCI as well as healthy controls. Patients with PD-CI (n, 27; mean age, 75.3 years; standard deviation (SD), 7.2; sex, 15 men) and PD-NCI (n, 34; mean age, 70.0 years; SD, 7.9; sex, 20 men) were recruited from the outpatient clinic of the Department of Neurology, National Taiwan University Hospital (NTUH), between December 2019 and December 2020. A total of 33 HCs (mean age, 65.2 years; SD, 5.6; sex, 17 men) who met the following inclusion criteria were enrolled: having MMSE (≥ 25) and MoCA (≥ 26) and not having any self-reported substance abuse, brain injury, severe ongoing health problems, and a history of neurological diseases or psychiatric disorders. The details of population characteristics have been provided in the manuscript.
Recruitment	Specifically for clinical patients, clinical diagnoses of PD were established using the criteria stipulated by the UK Brain Bank and were confirmed through Tc99m-TRODAT imaging performed by experienced neurologists from the PD center of NTUH. Patients with similar education levels and disease durations were preferably included whereas those with malignancies, autoimmune disorders, cerebrovascular disorders, major systemic diseases, self-reported substance abuse, brain surgery, or other known neuropsychiatric diseases were excluded. The patients' symptoms at initial recruitment were assessed using the Unified Parkinson's Disease Rating Scale (UPDRS) and Hoehn and Yahr (H&Y) scale. Cognitive function was first screened using the MoCA and Mini-Mental State Examination (MMSE). All patients underwent a complete NPT to confirm their cognitive impairment status including Wechsler Memory Scale (WMS) and Color Trails Test (CTT); the former was used to assess memory-related deficits, and the latter evaluated cognitive processing speed, attention, and executive function.
Ethics oversight	The Institutional Review Board of NTUH approved the study (No: 201904092RINC), and all participants provided written informed consent.

Note that full information on the approval of the study protocol must also be provided in the manuscript.

Field-specific reporting

Please select the one below that is the best fit for your research. If you are not sure, read the appropriate sections before making your selection.

☒ Life sciences ☐ Behavioural & social sciences ☐ Ecological, evolutionary & environmental sciences

For a reference copy of the document with all sections, see nature.com/documents/nr-reporting-summary-flat.pdf

Life sciences study design

All studies must disclose on these points even when the disclosure is negative.

Sample size	Patients with PD-CI (n, 27; mean age, 75.3 years; standard deviation (SD), 7.2; sex, 15 men), PD-NCI (n, 34; mean age, 70.0 years; SD, 7.9; sex, 20 men), and 33 HCs (mean age, 65.2 years; SD, 5.6; sex, 17 men) were recruited in this study. Sample size estimation was conducted in this study. To ensure the sufficient statistical power from the collected samples, we run the statistical power analysis for our main analysis (ANCOVA); it showed that the statistical power was 0.8207, which was an acceptable statistical power for our statistical inference.
Data exclusions	For clinical patients, clinical diagnoses of PD were established using the criteria stipulated by the UK Brain Bank and were confirmed through Tc99m-TRODAT imaging performed by experienced neurologists from the PD center of NTUH. Patients with similar education levels and disease durations were preferably included whereas those with malignancies, autoimmune disorders, cerebrovascular disorders, major

	systemic diseases, self-reported substance abuse, brain surgery, or other known neuropsychiatric diseases were excluded. The HCs who met the following inclusion criteria were enrolled: having MMSE (≥ 25) and MoCA (≥ 26) and not having any self-reported substance abuse, brain injury, severe ongoing health problems, and a history of neurological diseases or psychiatric disorders.
Replication	In the hierarchical analytic design, we replicated the findings in PD-CI and PD-NCI by performing the subgroup analysis (shown in the supplementary materials) to validate and confirm the validity of statistical results shown in the main text.
Randomization	This study enrolled the patients with PD-CI and PD-NCI as well as healthy controls. Patients with PD-CI and PD-NCI were recruited from the outpatient clinic of the Department of Neurology, National Taiwan University Hospital (NTUH) without randomization. To adjust the statistical bias from covariates, we compared the GM- and WM-PAD scores of the PD-CI, PD-NCI, and HC groups by performing analyses of covariance (ANCOVAs) adjusted for chronological age, sex, and education. A post hoc analysis achieved by ANCOVA was used to test between-group differences while adjusting age, sex, and education, and the Benjamini–Hochberg method was used to address the multiple-comparison problem. To further elucidate the potential influence of motor-specific symptoms on the observed disparity in PAD metrics between PD-CI and PD-NCI, a subgroup comparison was conducted with the inclusion of an additional covariate, the UPDRS part III (motor-specific symptom severity).
Blinding	The patients were recruited from the outpatient clinical and diagnosed by the neurologists and neuroradiologists. During this recruitment phase, the group assignment was not in a blind fashion. However, all images used in this study (i.e. neuroimaging data from PD-CI, PD-NCI, and HC) were analyzed together including image preprocessing, image feature extraction, and brain age prediction, without the annotation of group labels throughout the image analysis (i.e. blinding fashion). Thus, the technical batch effect of brain age metrics should not exist between groups.

Reporting for specific materials, systems and methods

We require information from authors about some types of materials, experimental systems and methods used in many studies. Here, indicate whether each material, system or method listed is relevant to your study. If you are not sure if a list item applies to your research, read the appropriate section before selecting a response.

Materials & experimental systems

n/a	Involved in the study
☒	☐ Antibodies
☒	☐ Eukaryotic cell lines
☒	☐ Palaeontology and archaeology
☒	☐ Animals and other organisms
☐	☒ Clinical data
☒	☐ Dual use research of concern
☒	☐ Plants

Methods

n/a	Involved in the study
☒	☐ ChIP-seq
☒	☐ Flow cytometry
☐	☒ MRI-based neuroimaging

Clinical data

Policy information about clinical studies

All manuscripts should comply with the ICMJE guidelines for publication of clinical research and a completed CONSORT checklist must be included with all submissions.

Clinical trial registration	This study did acquire clinical data for the observational study but did not involve clinical trials with interventions.
Study protocol	NA.
Data collection	This study enrolled the patients with PD-CI and PD-NCI as well as healthy controls. Patients with PD-CI (n, 27; mean age, 75.3 years; standard deviation (SD), 7.2; sex, 15 men) and PD-NCI (n, 34; mean age, 70.0 years; SD, 7.9; sex, 20 men) were recruited from the outpatient clinic of the Department of Neurology, National Taiwan University Hospital (NTUH), between December 2019 and December 2020. A total of 33 HCs (mean age, 65.2 years; SD, 5.6; sex, 17 men) who met the following inclusion criteria were enrolled: having MMSE (≥ 25) and MoCA (≥ 26) and not having any self-reported substance abuse, brain injury, severe ongoing health problems, and a history of neurological diseases or psychiatric disorders. The details of population characteristics have been provided in the manuscript.
Outcomes	This study was an observational study (no clinical trials and interventions were conducted and administered).

Plants

Seed stocks | NA

Novel plant genotypes | NA

Authentication | NA

Magnetic resonance imaging

Experimental design

Design type | No fMRI design was employed.

Design specifications | NA

Behavioral performance measures | NA

Acquisition

Imaging type(s) | T1-weighted images, diffusion spectrum imaging

Field strength | 3T

Sequence & imaging parameters | All brain images used in this study were acquired using a 3-Tesla MRI scanner (Tim Trio; Siemens, Erlangen, Germany) with a 32-channel phased-array head coil. We collected T1-weighted images and diffusion spectrum imaging (DSI) data sets to estimate GM and WM features, respectively. T1-weighted imaging was performed using a three-dimensional (3D) magnetization-prepared rapid gradient-echo sequence with the following parameters: repetition time/echo time (TR/TE) = 2000/3 ms; flip angle = 9°; field of view (FOV) = 256 × 192 × 208 mm^3; and isotropic spatial resolution = 1 mm^3. DSI was performed using a pulsed-gradient spin-echo echo-planar imaging sequence with the following parameters: bmax = 4000 s/mm^2; TR/TE = 9600/130 ms; slice thickness = 2.5 mm; FOV = 200 × 200 mm^2; and in-plane spatial resolution = 2.5 × 2.5 mm^2. The acquisition scheme comprised 102 diffusion-encoding gradients that corresponded to the Cartesian grids in the half-sphere of a 3D diffusion-encoding space and employed bipolar diffusion-encoding gradient design to minimize the eddy current artifact at the sequence level. Each MRI scanning process involved T1-weighted imaging (~3 min) and DSI (~16 min).

Area of acquisition | Whole brain scans were acquired.

Diffusion MRI ☒ Used ☐ Not used

Parameters | See above

Preprocessing

Preprocessing software | For structural analysis, all the structural and diffusion MRI data sets used in the present study exhibited satisfactory image quality. To extract GM features from the T1-weighted images, voxel-based morphometry and surface-based morphometry were performed using the Computational Anatomy Toolbox (CAT) (Gaser, C., Dahnke, R., Thompson, P. M., Kurth, F. & Luders, E. CAT-a computational anatomy toolbox for the analysis of structural MRI data. BioRxiv, 2022.2006. 2011.495736 (2022)). The details were provided in the main text and supplementary materials. For diffusion MRI data analysis, we used our well-developed algorithm that has been published and well-evaluated in our previous literature Chen, Y. J. et al. Automatic whole brain tract-based analysis using predefined tracts in a diffusion spectrum imaging template and an accurate registration strategy. Human brain mapping 36, 3441-3458 (2015). The details were provided in the main text and supplementary materials.

Normalization | See above

Normalization template | See above

Noise and artifact removal | See above

Volume censoring | NA

Statistical modeling & inference

Model type and settings: ANCOVA, multiple linear regression, partial correlation

Effect(s) tested: For ANCOVA, the group effect (PD-CI, PD-NCI, and HC) was tested. For multiple linear regression, the effects of brain age estimates with clinical factors were tested. For partial correlation, the correlation coefficients were tested.

Specify type of analysis: ☐ Whole brain ☒ ROI-based ☐ Both

Anatomical location(s): Voxel-based morphometry was applied to estimate regional volume features in accordance with the LONI probabilistic brain atlas, which contains 56 regions of interest (Shattuck, D. W. et al. Construction of a 3D probabilistic atlas of human cortical structures. Neuroimage 39, 1064-1080 (2008).). Thickness features were sampled using 68 cortical regions of interest defined by the Desikan–Killiany atlas (Desikan, R. S. et al. An automated labeling system for subdividing the human cerebral cortex on MRI scans into gyral based regions of interest. Neuroimage 31, 968-980 (2006)). For white matter features, we sampled tract-specific features according to 45 predefined tract bundle coordinates (Tseng, W. Y. I., Hsu, Y. C., Chen, C. L., Kang, Y. J., Kao, T. W., Chen, P. Y., & Waiter, G. D. (2021). Microstructural differences in white matter tracts across middle to late adulthood: a diffusion MRI study on 7167 UK Biobank participants. Neurobiology of Aging, 98, 160-172.).

Statistic type for inference (See Eklund et al. 2016): Brain age estimates were used to represent the whole brain integrity in this study.

Correction: Benjamini–Hochberg method was used to address the multiple-comparison problem in terms of the number of brain age metrics and the number of clinical measures.

Models & analysis

n/a	Involved in the study
☒	☐ Functional and/or effective connectivity
☒	☐ Graph analysis
☐	☒ Multivariate modeling or predictive analysis

Multivariate modeling and predictive analysis: Brain age prediction and classification were implemented and tested in this study. The details were provided in the main text and supplementary materials.
